# Pathological modeling of TBEV infection reveals differential innate immune responses in human neurons and astrocytes that correlate with their susceptibility to infection

**DOI:** 10.1186/s12974-020-01756-x

**Published:** 2020-03-03

**Authors:** Mazigh Fares, Marielle Cochet-Bernoin, Gaëlle Gonzalez, Claudia N. Montero-Menei, Odile Blanchet, Alexandra Benchoua, Claire Boissart, Sylvie Lecollinet, Jennifer Richardson, Nadia Haddad, Muriel Coulpier

**Affiliations:** 1 UMR1161 Virologie, Anses, INRAE, Ecole Nationale Vétérinaire d’Alfort, Université Paris-Est, Maisons-Alfort, France; 2 MRC-University of Glasgow Centre for Virus Research, Glasgow, Scotland UK; 3 CRCINA, UMR 1232, INSERM, Université de Nantes, Université d’Angers, F-49933 Angers, France; 4 Centre de Ressources Biologiques, CHU Angers, BB-0033-00038 Angers, France; 5 CECS, I-STEM, AFM, Evry, France; 6 UMR BIPAR 956, Anses, INRAE, Ecole Nationale Vétérinaire d’Alfort, Université Paris-Est, Maisons-Alfort, France

**Keywords:** Neurotropic virus, Flavivirus, Tick-borne encephalitis virus, Central nervous system, Human neuronal/glial cells, Viral tropism, Innate immunity, Neuropathogenesis, Pathological modeling

## Abstract

**Background:**

Tick-borne encephalitis virus (TBEV) is a member of the *Flaviviridae* family, *Flavivirus* genus, which includes several important human pathogens. It is responsible for neurological symptoms that may cause permanent disability or death, and, from a medical point of view, is the major arbovirus in Central/Northern Europe and North-Eastern Asia. TBEV tropism is critical for neuropathogenesis, yet little is known about the molecular mechanisms that govern the susceptibility of human brain cells to the virus. In this study, we sought to establish and characterize a new in vitro model of TBEV infection in the human brain and to decipher cell type-specific innate immunity and its relation to TBEV tropism and neuropathogenesis.

**Method:**

Human neuronal/glial cells were differentiated from neural progenitor cells and infected with the TBEV-Hypr strain. Kinetics of infection, cellular tropism, and cellular responses, including innate immune responses, were characterized by measuring viral genome and viral titer, performing immunofluorescence, enumerating the different cellular types, and determining their rate of infection and by performing PCR array and qRT-PCR. The specific response of neurons and astrocytes was analyzed using the same approaches after enrichment of the neuronal/glial cultures for each cellular subtype.

**Results:**

We showed that infection of human neuronal/glial cells mimicked three major hallmarks of TBEV infection in the human brain, namely, preferential neuronal tropism, neuronal death, and astrogliosis. We further showed that these cells conserved their capacity to mount an antiviral response against TBEV. TBEV-infected neuronal/glial cells, therefore, represented a highly relevant pathological model. By enriching the cultures for either neurons or astrocytes, we further demonstrated qualitative and quantitative differential innate immune responses in the two cell types that correlated with their particular susceptibility to TBEV.

**Conclusion:**

Our results thus reveal that cell type-specific innate immunity is likely to contribute to shaping TBEV tropism for human brain cells. They describe a new in vitro model for in-depth study of TBEV-induced neuropathogenesis and improve our understanding of the mechanisms by which neurotropic viruses target and damage human brain cells.

## Background

Tick-borne encephalitis virus (TBEV) belongs to the genus *Flavivirus* (family *Flaviviridae*), whose members include several important human pathogens transmitted by arthropods, such as Japanese encephalitis virus (JEV), West Nile virus (WNV), Zika virus (ZIKV), and Powassan virus (POWV). From a medical point of view, TBEV is the most important arbovirus in Europe and North-Eastern Asia. Its endemic zone spreads from Northern, Central, and Eastern Europe to Far East Asia [1]. It induces a range of symptoms from mild flu-like symptoms to severe encephalitis and paralysis, often with long-term neurological sequelae [2]. The incidence of the disease has increased in recent decades, and autochthonous cases are regularly reported in new areas of Western Europe, reflecting an expansion to non-endemic areas [3]. Despite commercialization of an effective vaccine [4], between 8000 and 13,000 annual cases of tick-borne encephalitis have been reported worldwide since the 1990s [5]. No therapy is currently available [6].

TBEV is usually transmitted to humans from infected ticks, mainly of the *Ixodes* family, but may occasionally be acquired by consumption of unpasteurized dairy products from infected livestock [7–9]. Upon inoculation into the human skin, initial infection and replication occur in local dendritic cells (DCs). DCs are believed to transport the virus to draining lymph nodes from which it spreads into the bloodstream and induces viremia. It may then cross the blood-brain barrier and cause widespread lesions in the brain. These include inflammatory changes, neuronal damage, and glial reactivity in several brain areas, including the spinal cord, brainstem, cerebellum, and striatum [10, 11]. Neurons are the primary target of infection [12], but other cells in the central nervous system (CNS) may contribute to TBEV-induced neuropathogenesis. Both infiltrating immunocompetent cells, mainly CD8^+^ T cells, and resident glial cells, such as astrocytes and microglial cells, have been shown to play a role [13, 14]. Neuronal damage may thus be mediated directly by viral infection or indirectly by infiltrating immunocompetent cells, inflammatory cytokines, and activated resident glial cells.

The innate immune response is the first line of defense against viral infection. Type I interferons (IFNs) are of particular importance in this process. Through binding to the IFN alpha/beta receptor (IFNAR), they act via autocrine or paracrine signaling [15–17] and trigger the activation of a large number of interferon-stimulated genes (ISGs) that can inhibit almost every step of the viral life cycle [18]. In recent years, it has become clear that parenchymal cells of the CNS play a major role in the development of the innate immune response and the protection of infected individuals after CNS infection [19–24]. Neurons and astrocytes are not passive targets, as they are known to produce and respond to type I IFNs. Nevertheless, the innate immune programs activated in these cell types during TBEV infection and their impact on viral tropism and neuropathogenesis remain poorly known.

Animal models have been widely used to elucidate the cellular and molecular mechanisms of TBEV-induced neuropathogenesis [2]. Nevertheless, the results obtained from such studies may be difficult to transpose to human neuropathogenesis, as human antiviral responses differ substantially from those of other mammalian species [25, 26]. The biological relevance of models based on human CNS cells is thus increasingly recognized. These include neuronal/glial cell lines, primary neuronal/glial cells from human fetuses, and more recently, neuronal/glial cells derived from fetal neural progenitors (hNPCs), embryonic (hESCs), or induced pluripotent stem cells (hiPSCs). While primary human CNS cells are physiologically more relevant than cell lines, their use is limited by the difficulty in gaining access to cell sources. On the other hand, neuronal/glial cultures derived from neural progenitors are not only physiologically relevant, but also have the advantage of being available on demand. In recent years, they have become important tools to study neurotropic viruses [27].

The goal of this study was, first, to set up and characterize a new in vitro model of TBEV infection using complex co-cultures of hNPC-derived neuronal/glial cells and, second, to decipher cell-specific anti-TBEV immunity in the human CNS and its relation to TBEV tropism and neuropathogenesis. We showed that in vitro TBEV infection mimics several hallmarks of in vivo infection, including marked neuronal tropism and neuronal death, limited astrocyte susceptibility, astrogliosis, and induction of an antiviral response. Moreover, we demonstrated differential qualitative and quantitative antiviral capacities in human neurons and astrocytes that are correlated with their susceptibility to TBEV infection. Finally, we showed that human astrocytes exert a protective effect on neighboring TBEV-infected neurons.

## Methods

### Ethics statement

Human fetuses were obtained after legal abortion with written informed consent from the patient. The procedure for the procurement and use of human fetal central nervous system tissue was approved and monitored by the “Comité Consultatif de Protection des Personnes dans la Recherche Biomédicale” of Henri Mondor Hospital, France. The cells are declared at the “Centre de Ressources Biologiques” of the University Hospital in Angers BB-0033-00038 with reference numbers at the Research Ministry: declaration no. DC-2011-1467 and authorization no. AC-2012-1507.

The rabbit immunization protocol complied with EU legislation (authorization 12/04/11-6 given by the ANSES/ENVA/UPEC ethical committee).

### Culture of human neural progenitor cells

Human neural progenitor cells (hNPCs) were prepared and cultured as previously described in [28, 29]. They are self-renewing cells and therefore may provide an unlimited source of material.

### Neuronal and glial differentiation

hNPCs were seeded on matrigel-coated plates at a density of 30,000 cells/cm^2^. Differentiation into a mixed population of neuronal and glial cells was induced 24 h after plating by replacing N2A medium with 1:1 N2A and NBC media (N2A: advanced Dulbecco’s modified Eagle medium-F12 supplemented with 2 mM L-glutamine, 0.1 mg/ml apotransferrin, 25 μg/ml insulin, and 6.3 ng/ml progesterone; NBC: neurobasal medium supplemented with 2 mM L-glutamine and B27 without vitamin A 1X—Invitrogen, Life Technologies) and withdrawing EGF (TEBU, France) and bFGF (TEBU, France). Differentiation conditions were maintained for 13 days with medium replacement twice a week, prior to infection. Twenty-four-well plates (IBIDI, #82406) were used for fluorescent immunostaining, and 6-well plates (Falcon) were used to prepare lysates for RNA analyses.

### Virus and infection

TBEV-Hypr strain was a kind gift from Dr. S. Moutailler (Maisons-Alfort, France). The strain was isolated in 1953 from the blood of a 10-year-old child in the Czech Republic, and the complete sequence was published in [30]. A working stock was generated in VERO cells (VERO-ATCC-CCL81) cultured in MEM medium (ThermoFisher) supplemented with 2% fetal bovine serum (FBS). Titer was estimated by plaque assay on VERO cells. Neuronal/glial cells differentiated for 13 days were infected with the virus (MOI 10^−2^) for 1 h at 37 °C. The inoculum was removed and cells were incubated in fresh N2A-NBC medium. Virus titers were estimated by endpoint dilution on VERO cells (TCID50). All procedures with infectious materials were performed under bio-safety level-3 (BSL-3) conditions.

### RNA isolation and qPCR

**Fig. 1:**
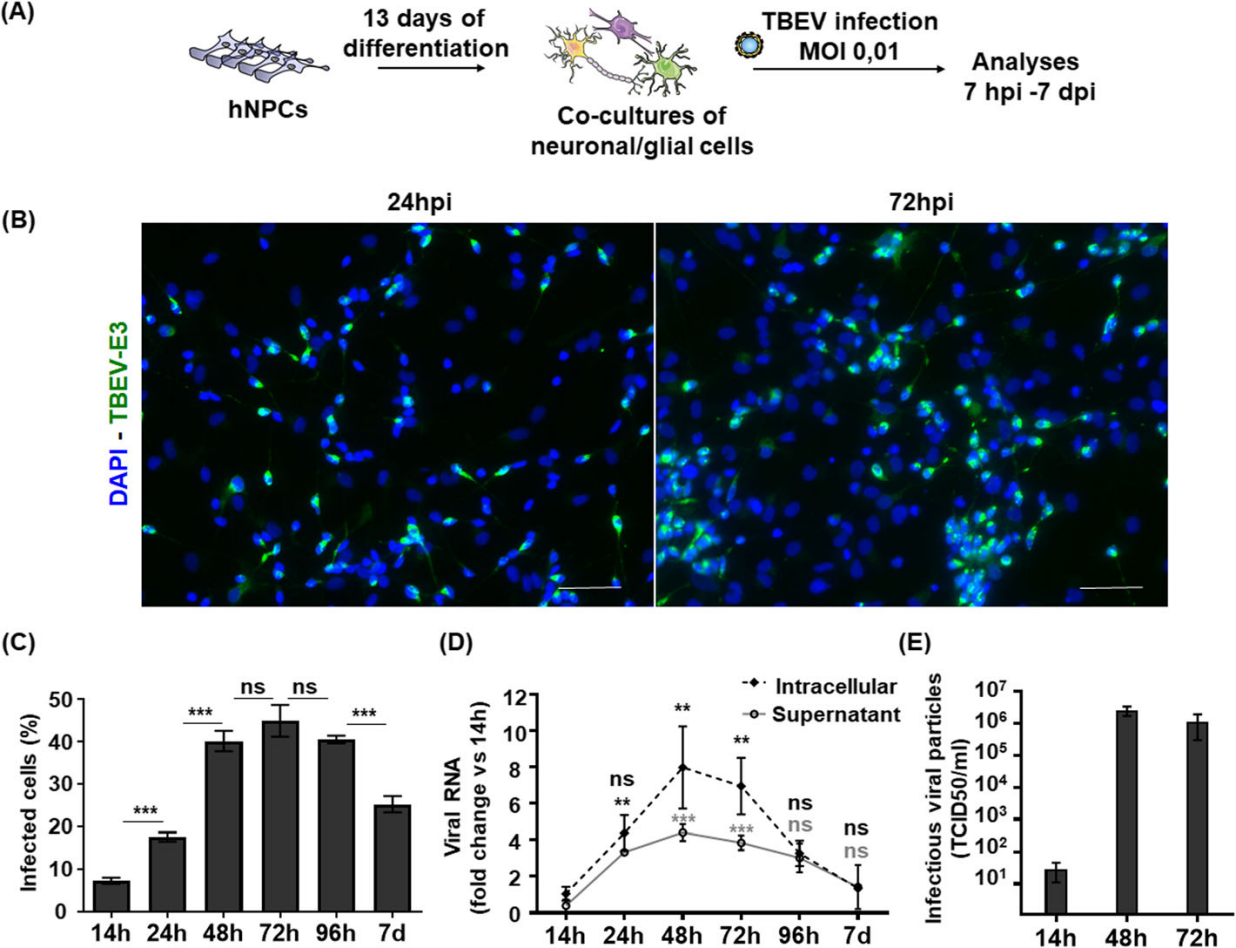
TBEV infects, replicates, and spreads in hNPC-derived brain cells. **a** Schematic representation of the experimental procedure. **b** Immunofluorescence labeling of differentiated hNPCs 24 h and 72 h following TBEV infection. An antibody directed against the domain 3 of the viral envelope (TBEV-E3, green) revealed infected cells. Nuclei were stained with DAPI (blue). **c** Enumeration of infected cells based on immunofluorescence labeling using an ArrayScan Cellomics instrument. **d** RNA from the supernatant and cell lysate of infected cells was analyzed by RT-qPCR to determine viral replication. **e** Supernatant was collected at 14, 48, and 72 hpi and titrated by endpoint dilution (TCID50) on VERO cells. Results are representative of 3 independent experiments performed in triplicate. Data are expressed as the mean ± SD. Statistical analysis was performed using one-way ANOVA (Bonferroni’s Multiple Comparison Test) with Graphpad Prism V6.0.1. ns, non-significant (*p* > 0.05); **p < 0.01, ***p < 0.001. Scale bar = 100 μm

RNA was isolated from infected and non-infected neuronal/glial co-cultures. Cells were lysed using the *NucleoMag® 96 RNA* kit (Macherey Nagel), and RNA was extracted with a *King Fisher Duo* automat (Fisher Scientific) following the manufacturer’s instructions. Extraction of viral RNA from supernatants of infected cells was performed using *QIAamp Viral RNA Mini Kit* (Qiagen) according to the manufacturer’s instructions. One hundred and sixty nanograms (Fig. 5) or 250 ng (Figs. 7 and 1d) of RNA from cell lysates and 2 μl of RNA from supernatant (Fig. 1d) were used to synthesize cDNA with the *SuperScript™ II Reverse Transcriptase* kit (ThermoFisher Scientific). Real-time PCR was performed using 2 μl of cDNA and *QuantiTect SYBR green PCR master* (Qiagen) with a LightCycler 96 instrument (Roche Applied Science), for a total volume of 20 μl of reaction mixture. For relative quantification, the − 2ΔΔCt method was used [31]. The reference genes were *GAPDH* or *HPRT1*. Primers pairs are listed in Additional file 1. TBEV primers pairs were from Schwaiger et al. [32].

### RT^2^ profiler PCR array

Equal volumes of RNA from biological triplicates were pooled for each condition. Two hundred to 500 ng of RNA were transcribed with the *RT*^*2*^*First Strand Kit* (SA Biosciences, Qiagen). Synthetized cDNA was subjected to a PCR array specific for the human antiviral response (*RT*^*2*^*Profiler PCR array*—PAHS-122Z, SA Biosciences, Qiagen), according to the manufacturer’s instructions. Data were normalized using the *HPRT1* house-keeping gene and analyzed with the − 2ΔΔCt method for relative quantification. According to the manufacturer’s instructions, an arbitrary cut-off of 3 was applied to determine significant differences. The analysis was performed using the Qiagen Data analysis center (http://www.qiagen.com/fr/shop/genes-and-pathways/data-analysis-center-overview-page/).

### Immunofluorescence assays and cell enumeration

Neuronal/glial cells were fixed for 20 min in 4% paraformaldehyde in PBS (Electron Microscopy Sciences) and standard immunofluorescence was performed using antibodies for HuC/HuD (Thermofisher #A21271), βIII-tubulin (Sigma #T8660), GFAP (Dako #M076101-2 or #Z033429-2), OLIG2 (R&D Systems #AF2418), and TBEV-E3. TBEV-E3 antibody was produced as follows: a rabbit polyclonal antiserum was raised against the TBEV E domain 3 after 3 sequential immunizations of 3-months old New Zealand rabbits with 100 μg of Ni2+-resins affinity-purified antigen adjuvanted with ISA50V2 (SEPPIC, France) at a 3-week interval and was collected on week 8 after the prime. The production of affinity-purified TBEV E domain 3 has been detailed in Beck et al. [33]. Cells were blocked for 1 h in 3% BSA (Sigma), 0.3% Triton-X-100 (VWR) in PBS 1X and primary antibodies were diluted in 1% BSA, 0.1% Triton-X-100 in PBS 1X, and incubated overnight at + 4 °C. Secondary antibodies were Alexa Fluor-488/546-conjugated anti-mouse/anti-rabbit IgG (Molecular Probes, Invitrogen). Nuclei were stained with 4′,6-diamidino-2-phenylindole (DAPI) (Life Technologies) at 0.1 ng/ml. For the assessment of apoptotic cell death, terminal deoxynucleotidyltransferase-mediated dUTP-biotin nick end labeling (TUNEL) staining was performed according to the manufacturer’s instructions (Promega, France). Cell sub-types and infected cells were enumerated either manually or automatically. For manual cell quantification (cells immunostained with antibodies directed against βIII-tubulin and GFAP), images were acquired with an *AxioObserver Z1* (Zeiss) inverted microscope using ZEN software (Zeiss) and analyzed using ImageJ 1.49 m software. For automated quantification (cells immunostained with antibodies directed against HuC/HuD, TBEV-E3 and OLIG2, neurites immunostained with an antibody against βIII-tubulin- and TUNEL-stained cells), images were acquired using the Cellomics ArrayScan automated microscope (Thermofisher Scientific) and analyzed using “Colocalization” or “Neuronal profiling” bio-applications on HCS Studio Cell Analysis Software V6.6.0 (Thermofisher Scientific). In all experiments, an average of 1200 (manual quantification) or 5000 (automated quantification) cells per well were enumerated. The digitized images shown were adjusted for brightness and contrast using ImageJ, without further alteration.

### Magnetic-activated cell sorting

Neuronal/glial cells differentiated for 13 days were detached using Gibco™ TrypLE™ Select Enzyme (1X) and collected into N2A-NBC medium. After centrifugation at 80×*g* for 10 min, cells were either sub-cultured (Uns-C) or supplemented with kynurenic acid buffer and sorted according to the manufacturer’s instructions using the Microbead Kit (Miltenyi Biotec #130–095-826). In brief, resuspended cells were incubated for 10 min at 4 °C with 20 μl biotin-conjugated anti-GLAST (ACSA-1) antibodies per 10^7^ cells, washed, and incubated with anti-biotin MicroBeads for 15 min at 4 °C. Cell sorting was performed using MS columns (Miltenyi Biotec, #130-042-201) placed in a MiniMACS™ separator (Miltenyi Biotec #130-090-312). The cell fractions found in the flow-through or bound to beads were composed of enriched neurons (En-N) and enriched astrocytes (En-As), respectively. Both sorted and unsorted cells were seeded at a density of 100,000 cells per cm^2^ on 24-well μ-plates (IBIDI, #82406) in N2A-NBC or conditioned medium (1:1, fresh N2A-NBC: supernatant of non-infected co-cultures differentiated for 13 days, conditioned for 48 h). Conditioned medium allowed neuronal survival in En-N cultures. Half of the medium was replaced every other day.

### Statistical analyses

Data are represented as mean ± standard deviation (SD). Statistical analyses were performed with GraphPad Prism V4.03 or V6.0.1 using an unpaired Student’s *t* test or a one-way ANOVA analysis (Bonferroni’s multiple comparison test), *p < 0.05, **p < 0.01, ***p < 0.001, non-significant (ns) = *p* > 0.05.

## Results

### TBEV infects brain cells differentiated from human fetal neural progenitors

HNPCs and their derived neuronal/glial cells were previously set up in our laboratory for the study of Borna disease virus, a neurotropic virus that belongs to the *Bornaviridae* family [28, 29]. Here, we used the same hNPCs prepared according to the experimental steps summarized in Fig. 1a. HNPCs were differentiated for 13 days, by which time all neurons were generated [29], before infection with the TBEV-Hypr strain at MOI 10^−2^. Infected neuronal/glial co-cultures were then analyzed over time. We first examined the capacity of the virus to infect, replicate, and disseminate within the co-culture. Examination of cells immunostained with an antibody specific for domain 3 of the TBEV envelope protein (TBEV-E3), at 24 and 72 h post-infection (hpi), revealed that TBEV entered human brain cells and spread within the co-culture (Fig. 1b). Enumeration of infected cells from 14 hpi to 7 dpi showed that 7.3 ± 0.7% of cells were indeed infected at 14 hpi, whereas 45.0 ± 4.0% were infected at 72 hpi, at the peak of infection (Fig. 1c). At a later time, 7 dpi, the number of infected cells decreased. A similar pattern of infection was observed when the viral RNA was quantified by RT-qPCR, whether in the supernatant or intracellularly, from 14 hpi to 7 dpi (Fig. 1d), with an increase in viral RNA observed up to 48–72 hpi followed by a decrease from 96 hpi to 7 dpi. This confirmed active replication of the virus in hNPC-derived brain cells. Quantification of viral titer by endpoint dilution further showed that infectious particles were released into the supernatant and increased from 14 hpi to 48 hpi and 72 hpi (Fig. 1e). Thus, the infection, replication and dissemination of virus were efficient in hNPC-derived neuronal/glial cells.

### TBEV infects human neurons, astrocytes, and oligodendrocytes

We next sought to determine which neural subsets were infected by TBEV in neuronal/glial co-cultures. We had previously shown that, upon growth factor withdrawal, hNPCs differentiated into GABAergic neurons and astrocytes [28, 29]. Oligodendrocytes, the third cell type that can be generated by differentiation of hNPCs, were not taken into consideration. Of note, microglial cells, which are of mesodermic origin, cannot be generated from fetal neural progenitor cells. To gain in precision, in the present study, we enumerated all 3 cell types, based on immunofluorescence staining (see Additional file 2A), 13 and 21 days after the onset of differentiation. Automatic enumeration of immunostained cells with antibodies directed against HuC/HuD (nuclear markers for neurons) and OLIG2 (nuclear marker for oligodendrocytes) revealed a population composed of 77.0 ± 3.2% (d13) and 74.1 ± 5.4% (d21) neurons and 1.4 ± 1.0% (d13) and 3.7 ± 1.0% (d21) oligodendrocytes (see Additional file 2B). Due to technical limitations (GFAP localization in astrocytic outgrowths and unavailability of a nuclear marker), astrocytes could not be automatically enumerated. The remaining population, namely total cells minus neurons and oligodendrocytes, comprising 21.5 ± 4.1% (d13) and 22.2 ± 4.4% (d21) of cells, was therefore considered to be composed of astrocytes (see Additional file 2B). The reliability of this enumeration procedure was confirmed by manual enumeration, as 22.8 ± 5.6% (d13) and 32.7 ± 5.0% (d21) of astrocytes were found using this method (see Additional file 2C). Thus, we confirmed that neurons and astrocytes were the major cell types in our co-cultures and showed that oligodendrocytes constituted less than 5% of the total cell population.

**Fig. 2:**
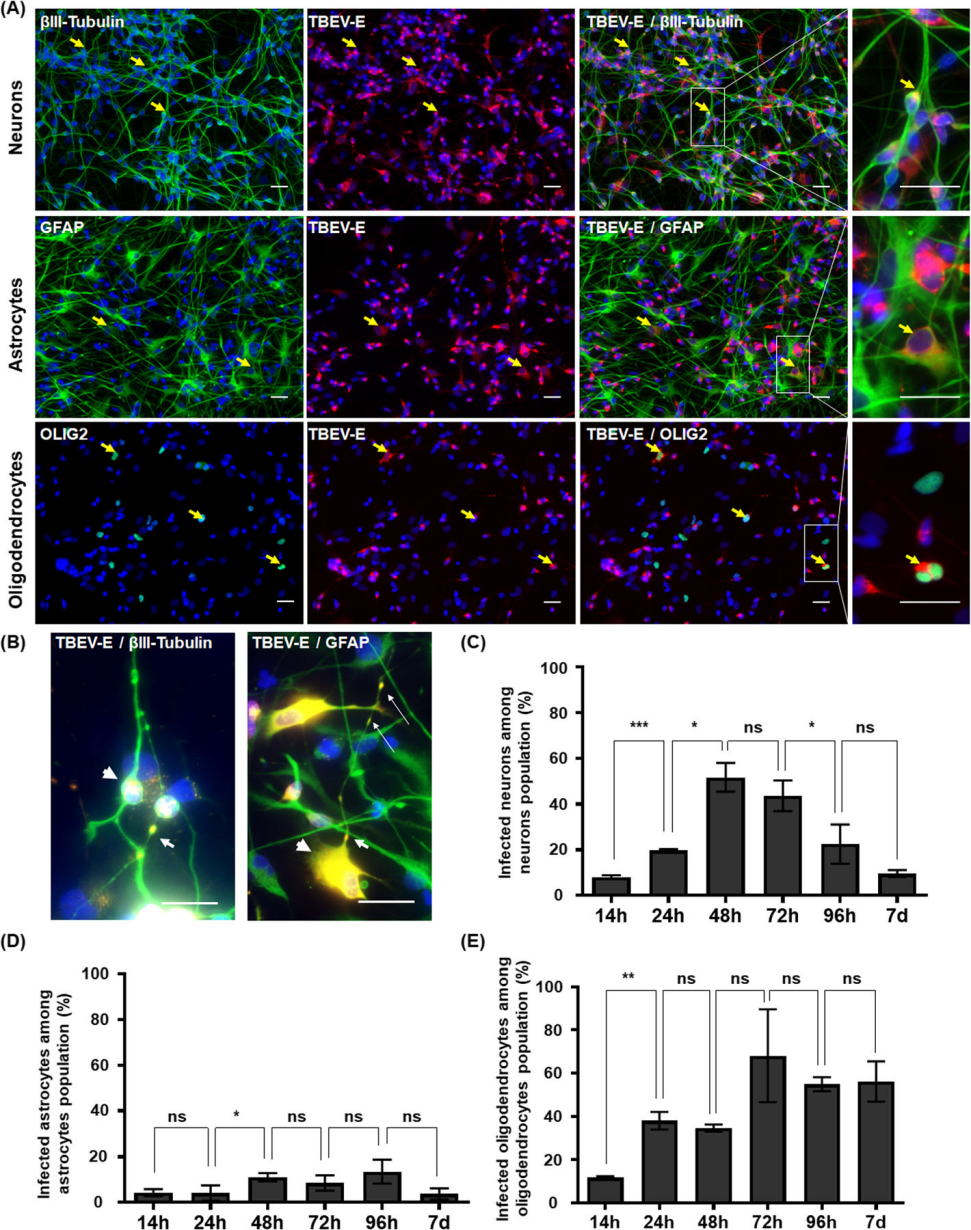
TBEV tropism for hNPC-derived brain cells. HNPCs differentiated for 13 days were infected with TBEV-Hypr at MOI 10^−2^. **a** Immunofluorescence labeling of infected cells at 24 hpi. Antibodies against βIII-tubulin (neurones), GFAP (astrocytes), or OLIG2 (oligodendrocytes) (green), and TBEV-E3 (red) were used. Nuclei were stained with DAPI (blue). Yellow arrows show infected neurons, astrocytes, and oligodendrocytes. Digital higher magnification is shown in the right panel. Oligodendrocytes were recolored from gray to green. Scale bar = 20 μm. **b** Higher magnification (digitally cropped) showing the viral envelope in perinuclear areas (arrowhead) and neurites and astrocytic outgrowths (arrows). Scale bar = 20 μm. **c**–**e** Percentage of infected cells based on immunofluorescence labeling during the course of infection for (**c**) neurons, (**d**) astrocytes, and (**e**) oligodendrocytes. Results are representative of at least 2 independent experiments performed in triplicate. Data are expressed as the mean ± SD. Statistical analysis was performed using a two-tailed unpaired *t* test with Graphpad Prism V6.0.1. ns, non-significant (*p* > 0.05); *p < 0.05, **p < 0.01, ***p < 0.001

To characterize TBEV cellular tropism, we infected hNPC-derived neuronal/glial co-cultures and followed infection from 14 h to 7 days. Cells were co-immunostained with antibodies directed against TBEV-E3 (infected cells) and βIII-tubulin or HuC/HuD (neurons), GFAP (astrocytes) and OLIG2 (oligodendrocytes). At 24 hpi, the 3 cell types were infected, as shown in Fig. 2a. Viral envelope strongly accumulated in the perinuclear region of the cytoplasm in all cell types. The protein could also be evidenced in certain neurites and astrocyte outgrowths, albeit with a lower intensity (Fig. 2b). We then sought to determine whether the virus spread within each cellular subpopulation. We therefore quantified infection in each cell type, at different time points during the course of the study (Fig. 2c–e). The general profile of infection at onset was similar in the 3 cell types, with an increase in the first days of infection up until a peak occurring at 48–72 hpi. While this phase was followed by a decrease from 96 hpi onwards in the neuronal population, the percentage of infection remained stable in astrocytes and oligodendrocytes, at least up to 7 dpi. Early in infection, at 14 hpi, a minority of cells were infected within each subset, namely 7.9 ± 1.2% of neurons, 4.3 ± 1.5% of astrocytes, and 11.7 ± 0.8% of oligodendrocytes. Later, however, at the peak of infection, the proportion of infected cells was high in neurons (55.2 ± 3.8%) and oligodendrocytes (68.0 ± 21.5%) but much lower in astrocytes (13.6 ± 5.3%), revealing differential propagation of the virus within the three sub-populations. Thus, whereas human neurons and oligodendrocytes were highly susceptible to TBEV infection, human astrocytes were more resistant.

### TBEV induces death of neurons and astrocytes

**Fig. 3:**
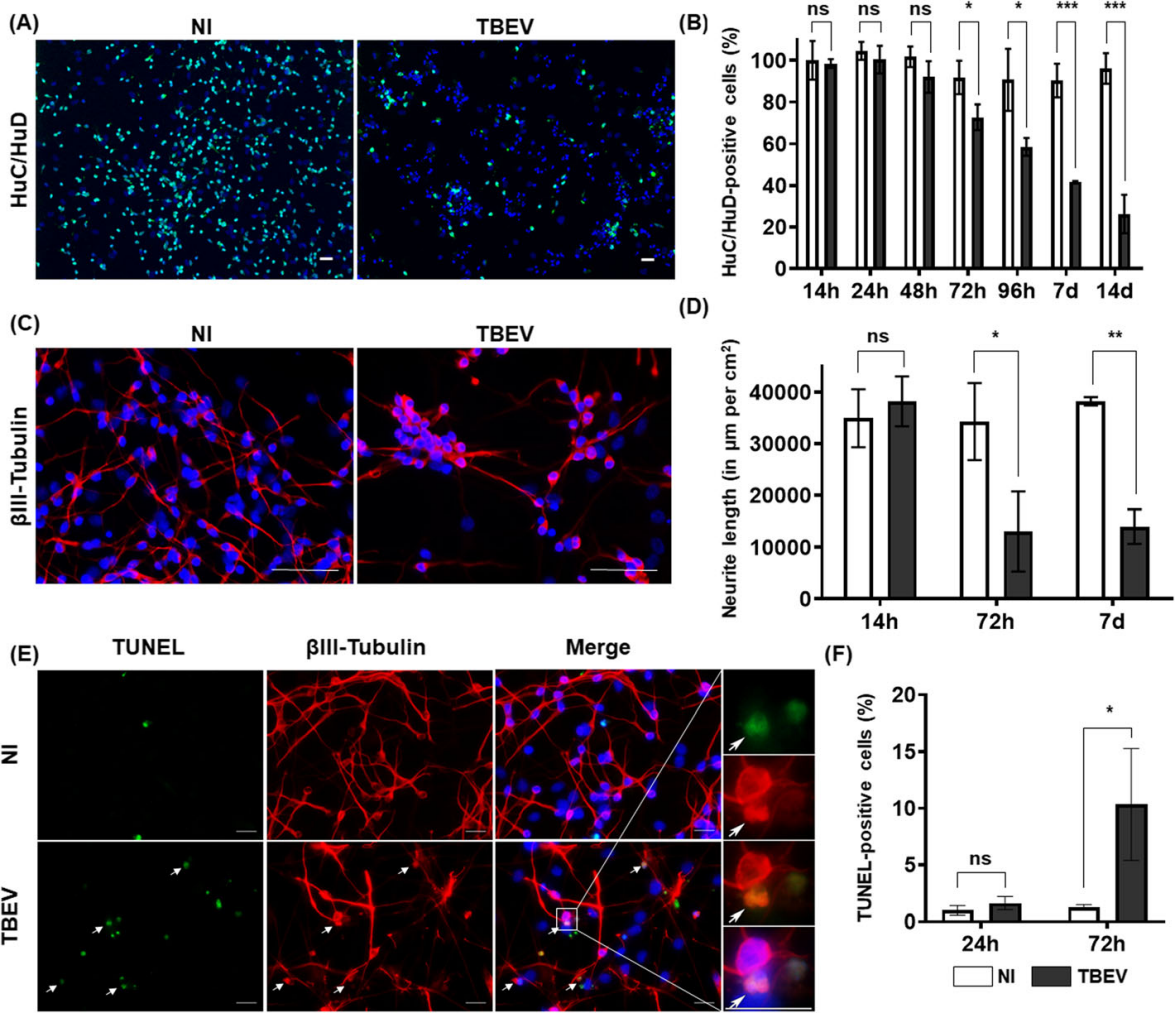
TBEV damages human neurons. HNPCs were differentiated for 13 days and infected with TBEV-Hypr at MOI 10^−2^. **a** Cells in non-infected (NI) and infected (TBEV) co-cultures were immunostained with an antibody directed against HuC/HuD (neurons, green) at 14 dpi. Nuclei were counterstained with DAPI. Scale bars = 100 μm. **b** Enumeration of HuC/HuD-positive cells using an ArrayScan Cellomics instrument. Normalization to non-infected HuC/HuD-positive cells at 14 hpi. **c** Cells in non-infected and infected cultures were immunostained with an antibody against βIII-tubulin (neurons, red) at 7 dpi. Note the paucity of neurites in TBEV-infected co-cultures. Scale bars = 100 μm. **d** Quantification of neurite network density (neurite length per square millimeter) using an ArrayScan Cellomics instrument. **e** Cells in non-infected and TBEV-infected cultures were TUNEL stained at 72 hpi. Note that TUNEL staining matches with βIII-tubulin immunostaining (arrows). Right panel show an apoptotic neuron (co-localization of βIII-tubulin and TUNEL staining). Scale bars = 20 μm. (**f**) Percentage of apoptotic cells based on TUNEL staining at 24 and 72 hpi. Results in **b**, **d**, and **f** are expressed as the mean ± SD and are representative of four (**b**) and two (**d**, **f**) independent experiments performed in triplicate, respectively. Statistical analysis was performed using a two-tailed unpaired *t* test with Graphpad Prism V6.0.1. ns, non-significant (*p* > 0.05); *p < 0.05, **p < 0.01, ***p < 0.001

As hNPC-derived neuronal/glial cells were highly infected, we then sought to evaluate whether TBEV induced cellular damage. Cultures were infected, and cells were fixed at several time points from 14 hpi to 14 dpi before immunostaining with antibodies specific for neuronal and glial cells, as previously described. We first examined the neuronal population. At 14 dpi, examination of HuC/HuD immunostaining revealed that TBEV-infected co-cultures were strongly depleted in neurons, as compared with their non-infected matched controls (Fig. 3a). Enumeration showed that neuronal survival was unaffected in the first days of infection (from 14 to 48 hpi) but confirmed that neuronal loss occurred as early as 72 hpi (25.1 ± 5.4% loss) and steadily increased from this point on, reaching 72.0 ± 10.3% at 14 dpi, the latest time point of our study (Fig. 3b). We then examined neuronal morphology based on βIII-tubulin immunostaining. At 7 dpi, a striking loss of neurites was observed in TBEV-infected cultures as compared with their non-infected matched controls (Fig. 3c). Quantification of total neurite length confirmed their loss not only at 7 dpi (76.1 ± 21.6% decrease), but also at 72 hpi (62.0 ± 22.6% decrease), whereas they were unaffected at an earlier time point (14 hpi) (Fig. 3d). Of note, whereas neuronal death became progressively more pronounced between 72 hpi and 7 dpi, neurite loss was as high at 72 hpi as at 7 dpi, suggesting that neurites alteration precedes neuronal death. In an attempt to determine the molecular mechanisms responsible for neuronal loss, we performed TUNEL staining at 24 and 72 hpi. Whereas there was no difference at 24 hpi, an increase in TUNEL staining was observed at 72 hpi in TBEV-infected cells as compared with their matched non-infected controls (Fig.3e, f), revealing that apoptotic events occurred as early as 72 hpi. Co-immunostaining with anti-βIII-tubulin antibody further demonstrated that apoptotic cells were neurons (Fig. 3e, right panel). Taken together, these results showed that TBEV infection strongly impaired neuronal survival in the co-cultures and, moreover, suggested that neurite alteration preceded neuronal death. They also explained the decrease in the percentage of infected cells and viral RNA at 7 dpi as shown in Fig. 1c/d. Indeed, as neurons represented the most numerous and highly infected cells, their massive death by that time point would have inevitably diminished the percentage of total infected cells.

**Fig. 4:**
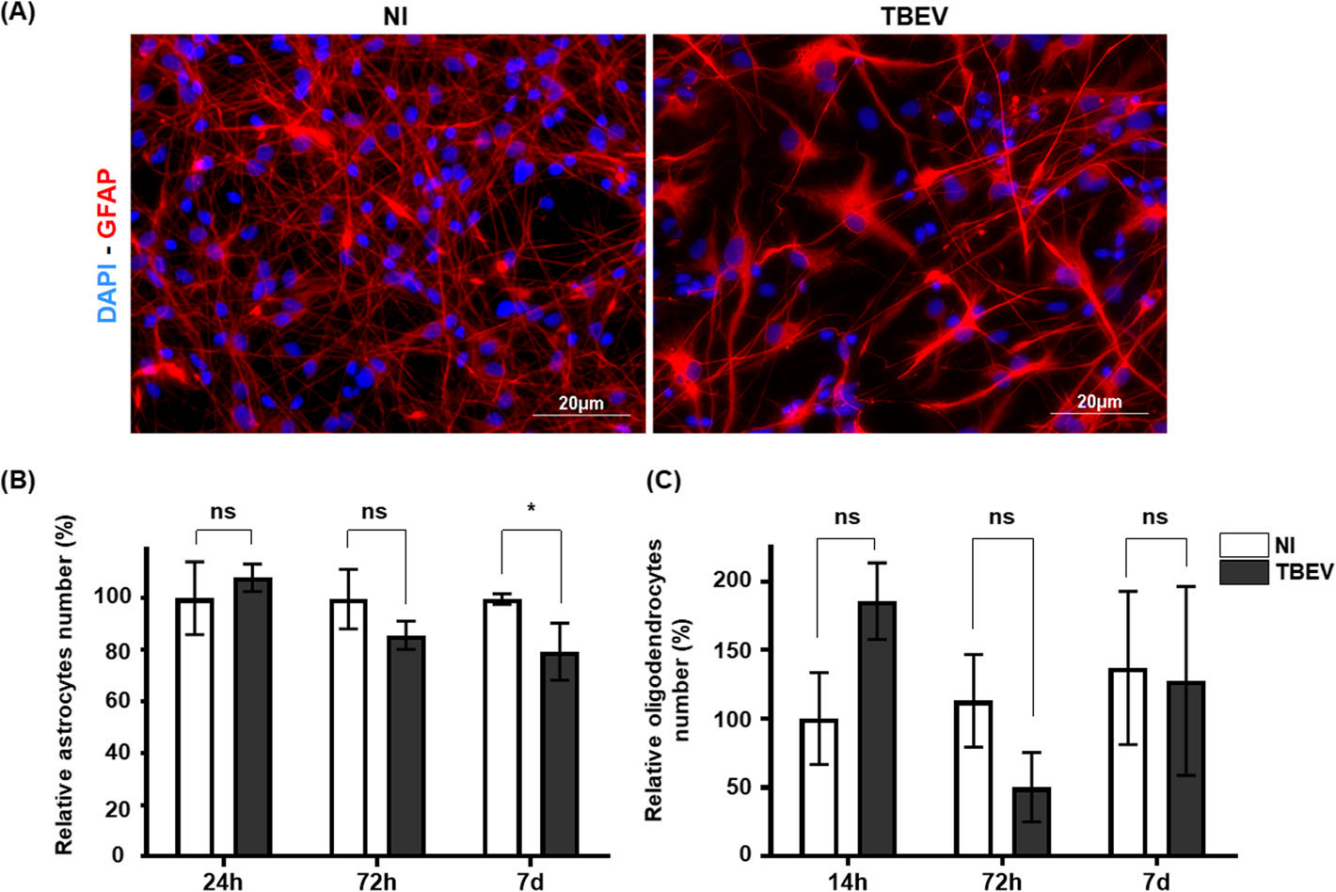
Impact of TBEV on human glial cells. HNPCs were differentiated for 13 days and infected with TBEV-Hypr at MOI 10^−2^. **a** Cells in non-infected (NI) and infected (TBEV) co-cultures were immunostained with an antibody directed against GFAP (astrocytes, red) at 7 dpi. Nuclei were counterstained with DAPI. Scale bars = 20 μm. **b** Manual enumeration of GFAP-positive cells using ImageJ software. Normalization was performed relative to non-infected GFAP-positive cells at 24 hpi. **c** Cells immunostained with OLIG2 antibody were enumerated automatically. Normalization was performed relative to non-infected OLIG2-positive cells at 24 hpi. The results are expressed as the mean ± SD and are representative of two (oligodendrocytes) and three (astrocytes) independent experiments performed in triplicate. Statistical analysis was performed using a two-tailed unpaired *t* test with Graphpad Prism V6.0.1. ns, non-significant (*p* > 0.05); *p < 0.05

We next evaluated whether glial cells were damaged. Examination of astrocytes immunostained with an antibody directed against GFAP at 7 dpi revealed hypertrophic cells in TBEV-infected cultures, as compared with their non-infected matched controls (Fig. 4a). This change in morphology is reminiscent of astrogliosis, a common feature of stressed astrocytes. Enumeration of GFAP-positive cells was then carried out at 24 hpi, 72 hpi, and 7 dpi. Their number was not significantly altered at the earlier time points, 24 hpi and 72 hpi, but a decrease of 20.7 ± 11.1% was observed at 7 dpi compared with non-infected matched controls (Fig. 4b). Thus, TBEV infection diminished survival of not only neurons but also astrocytes, although in a more moderate manner for the latter. By contrast, enumeration of OLIG-2-positive cells did not reveal a significant difference in oligodendrocyte number in TBEV-infected and non-infected cultures (Fig. 4c), showing that despite direct TBEV infection, survival of oligodendrocytes was unaffected. Taken together, our results demonstrated that subsets of hNPC-derived brain cells, that is, neurons, astrocytes, and oligodendrocytes, were differentially affected by TBEV infection. In particular, neurons were highly susceptible as regards both infection and mortality, whereas astrocytes were more resistant. Oligodendrocytes were susceptible to infection, but their survival was unaffected.

### Human NPC-derived neuronal/glial co-cultures develop a strong antiviral response to TBEV infection

**Fig. 5:**
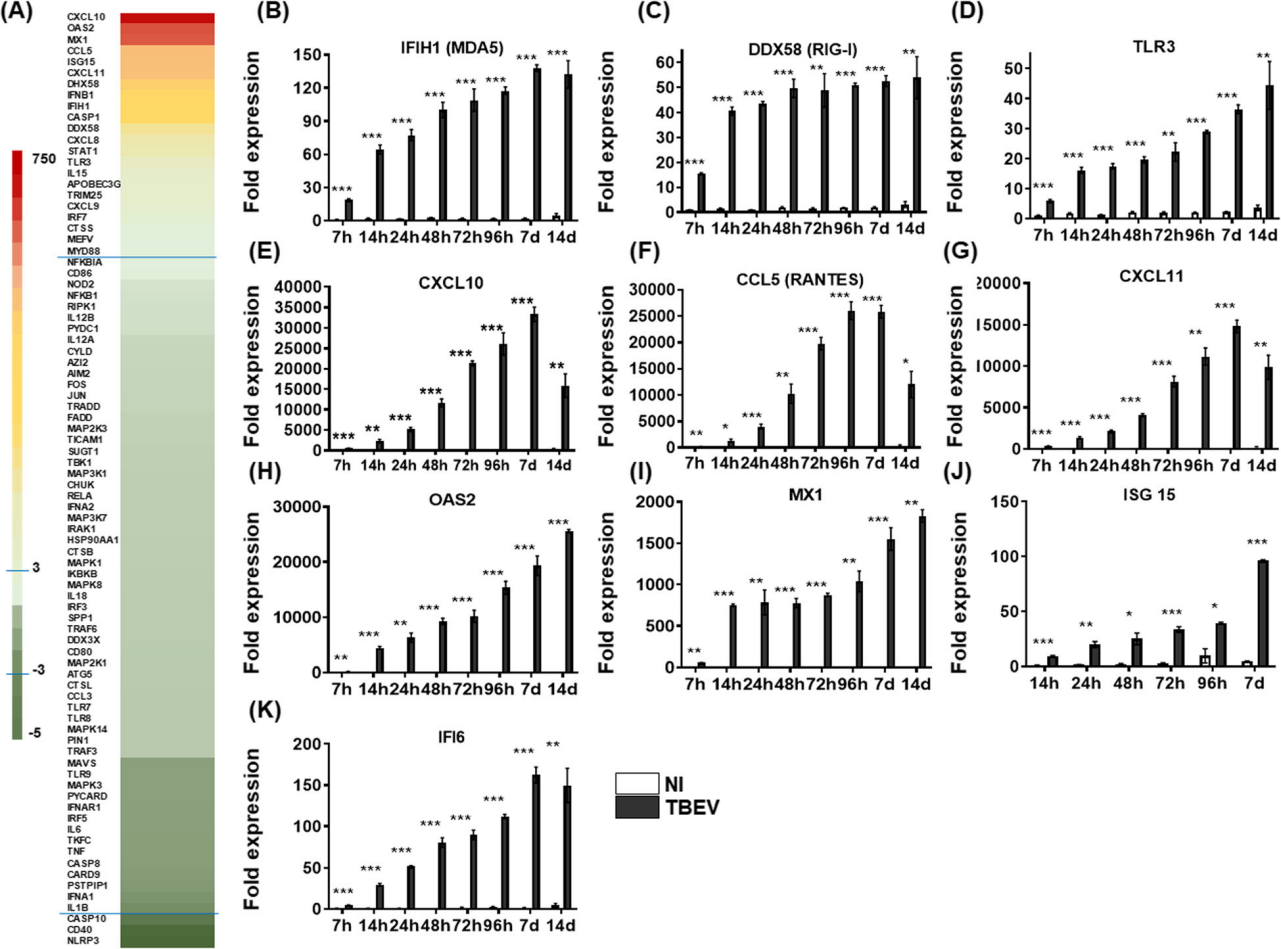
TBEV-induced antiviral response in hNPC-derived neuronal/glial cells. **a** TBEV-infected neuronal/glial cells (MOI 10^−2^) and their matched NI controls were analyzed 24 hpi using an RT^2^ Profiler PCR array specific for the human antiviral response. The heat map shows the differential expression of 84 analyzed human genes. The most highly up- and downregulated genes are colored in red and dark green, respectively. The blue lines indicate the arbitrary cut-off of 3. Genes between the two lines are considered nonregulated. **b**–**k** RT-qPCR analyses of selected antiviral genes. Gene expression was normalized to *HPRT1* gene, and the − 2ΔΔCt method was used for relative quantification (normalization to non-infected cells at 7 hpi). Data are expressed as the mean ± SD. Results are representative of one experiment performed on pooled triplicates (PCR array) or two independent experiments performed in triplicate (qPCRs). Statistical analysis was performed using a two-tailed unpaired *t* test with Graphpad Prism V6.0.1. ns, non-significant (*p* > 0.05); *p < 0.05, **p < 0.01, ***p < 0.001

When infected with virus, cells initiate an antiviral response that aims at controlling viral replication. In order to determine whether the human neuronal/glial cells used in our study had conserved the capacity to develop such a response upon TBEV infection, we analyzed the differential expression of 84 human genes involved in the antiviral response, using a PCR array approach. Transcripts from hNPC-derived neuronal/glial cells infected with TBEV for 24 h were pooled from biological triplicates and compared with transcripts from their matched non-infected controls. The studied genes are shown in Fig. 5a and Additional file 3. After applying an arbitrary cut-off of threefold, 25 genes were shown to be significantly modulated in TBEV-infected cells, among which 22 genes were upregulated and 3 were downregulated (Fig. 5a). The former category included pathogen recognition receptors (PRRs), cytokines, including IFN-β, and ISGs. Upregulation of nine of these genes, 3 PRRs—IFIH1/MDA5 (Fig. 5b), DDX58/RIG-I (Fig. 5c), and TLR3 (Fig. 5d); 3 pro-inflammatory cytokines—CXCL10 (Fig. 5e), CCL5/RANTES (Fig. 5f), and CXCL11 (Fig. 5g); and 3 ISGs—OAS2 (Fig. 5h), MX1 (Fig.5i), and ISG15 (Fig. 5j), was confirmed using RT-qPCR. IFI6 (Fig. 5k), an additional ISG that was recently shown to protect cells from *Flavivirus* infection [34], was also shown to be upregulated. For most of these genes, kinetic analyses further revealed that their expression was activated as early as 7 hpi and progressively increased during the course of infection up to 14 dpi, with the exception of pro-inflammatory cytokines whose expression abruptly decreased at 14 dpi (Fig. 5e–g). The latter, however, remained highly upregulated, as compared with their matched non-infected controls. These data indicated that TBEV-infected hNPC-derived neuronal/glial cells had the capacity to respond to TBEV infection by developing a strong and lasting antiviral response.

### Differential antiviral response in human neurons and human astrocytes

**Fig. 6:**
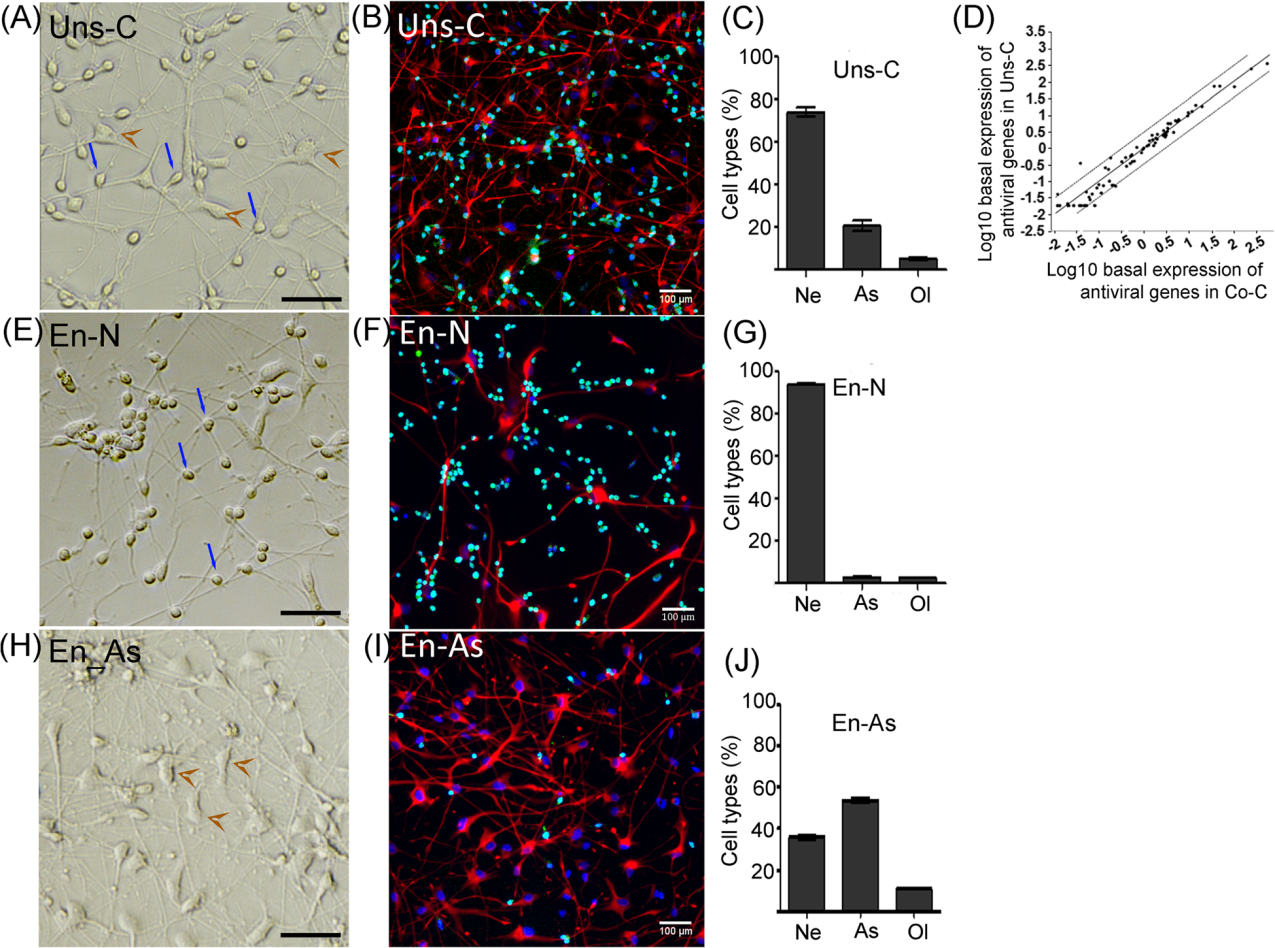
Enrichment of human neurons and astrocytes by magnetic-activated cell sorting. HNPC-derived neuronal/glial cells differentiated for 13 days were sorted using MACS technology. **a**, **e**, **h** Phase-contrast micrographs, showing Uns-C (**a**), En-N (**e**), and En-As (**h**), were acquired 96 h after re-seeding. Blue arrows indicate neurons and brown arrowheads indicate astrocytes. Scale bars, 50 μm. **b**, **f**, **i** Immunofluorescence staining with antibodies directed against neurons (HuC/HuD, green), astrocytes (GFAP, red), and oligodendrocytes (OLIG2, gray) markers showing the mixed and enriched population. DAPI is in blue. Scale bars = 100 μm. **c**, **g**, **j** Enumeration of neurons (Ne), astrocytes (As), and oligodendrocytes (Ol) in Uns-C (**c**), En-N (**g**), and En-As (**j**) based on immunofluorescence staining and using an ArrayScan Cellomics instrument. Data are representative of 4 independent experiments performed in triplicate. **d** Scatterplot of basal level of antiviral response genes in unsorted cells (Uns-C) compared with non-trypsinized cells (Co-C). Analysis was performed using an antiviral response PCR array. Genes along the black line have similar expression levels in the two cultures. Dotted lines represent an arbitrary cut-off of 3. Data are from a single experiment performed with pooled triplicates

Neurons and astrocytes are both known to participate in the antiviral response in the CNS [20, 35]. As regards oligodendrocytes, little is known so far [36]. Our results, showing high susceptibility of neurons but resistance of astrocytes to TBEV infection, led us to hypothesize that differences in their intrinsic capacity for antiviral defense might underlie their differential susceptibility. In order to test this hypothesis and decipher cell-autonomous anti-TBEV innate immunity in the human CNS, we sought to obtain cultures enriched in neurons (henceforth called En-N) or astrocytes (henceforth called En-As) and to compare their antiviral response. Oligodendrocytes were not considered further in this study, as their low number in our cultures precluded enrichment. After differentiation of hNPCs for 13 days, neuronal/glial cells were trypsinized and either directly re-seeded (unsorted cultures henceforth called Uns-C) or enriched for neurons (En-N) or astrocytes (En-As). We showed that the splitting procedure did not alter the neuronal/glial co-cultures (Uns-C). Indeed, 4 days after re-seeding, phase-contrast microscopy of Uns-C revealed typical neuronal (small sized with neurites) and astroglial (larger, flat, with outgrowths) cells (Fig. 6a), as typically observed in non-trypsinized co-cultures (henceforth called Co-C cells). Immunofluorescence staining using antibodies directed against neurons (HuC/HuD), astrocytes (GFAP), and oligodendrocytes (OLIG2) markers showed a mixed population (Fig. 6b) composed of 74.1 ± 4.1% neurons, 20.8 ± 4.9% astrocytes, and 5.1 ± 1.2% oligodendrocytes (Fig. 6c) revealing that the percentage of each subpopulation was unchanged in Uns-C as compared with Co-C cells. Basal expression of antiviral genes was also unchanged, as shown by the analysis of 84 genes of the antiviral response in Uns-C and Co-C cells (Fig. 6d). Enrichment in neurons and in astrocytes was confirmed by phase-contrast microscopy (Fig. 6e and h, respectively) and immunofluorescence staining (Fig. 6f and i, respectively) combined with cell enumeration showing that the En-N population was composed of 94.1 ± 0.4% neurons, 3.1 ± 0.4% astrocytes and 2.8 ± 0.2% oligodendrocytes (Fig. 6g) while the En-As population comprised 53.5 ± 2.7% astrocytes, 35.7 ± 2.8% neurons, and 10.8 ± 0.5% oligodendrocytes (Fig. 6j).

**Fig. 7:**
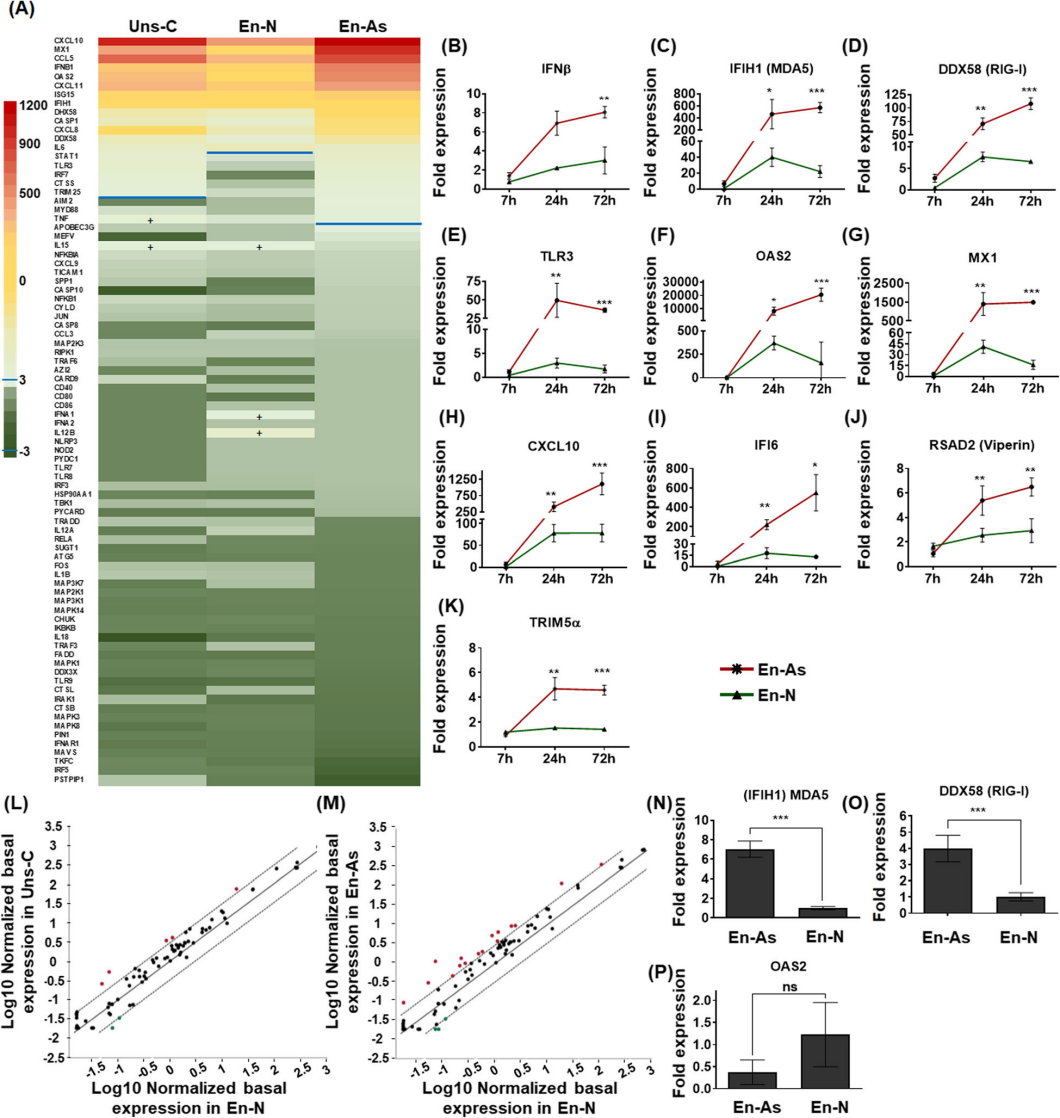
Antiviral response in enriched neurons, enriched astrocytes, and unsorted cells. **a**–**i** Expression of antiviral genes upon TBEV infection. **a** TBEV-infected Uns-C, En-N, and En-As (MOI 10^−2^) and their matched non-infected controls were analyzed 24 hpi using an RT^2^ profiler PCR array specific for the human antiviral response. Heat map showing the 84 human genes analyzed and their differential expression. Color code and blue line are as in Fig. 5. Note that genes noted (+) are above the cut-off of 3. **b**–**k** RT-qPCR analyses of selected antiviral response genes in En-As (red) and En-N (green). **l**–**p** Basal expression of antiviral genes. **l**, **m** Scatterplots of basal expression levels of antiviral response genes in enriched neurons (En-N) compared with that of unsorted cells (Uns-C) (**l**, **k**) or enriched astrocytes (En-As) (**m**). Analysis was performed using an antiviral response PCR array. **n**–**p** RT-qPCR analysis of the basal expression of IFIH1/MDA5 (**n**), DDX58/RIG-I (**o**), and OAS2 (**p**) genes in En-N and En-As. Gene expression was normalized to HPRT1 and the − 2ΔΔCt method was used for relative quantification (normalization to non-infected En-N, at 7hpi for **b**–**k**). The results are expressed as the mean ± SD. Data are representative of two independent experiments performed in triplicate (**b**–**k**, **n**–**p**) and one experiment performed with pooled triplicates (**l**, **m**). Statistical analyses comparing En-As and En-N were performed with Graphpad Prism V6.0.1 using a two-tailed unpaired *t* test. ns, non-significant (*p* > 0.05); *p < 0.05, **p < 0.01, ***p < 0.001

We then sought to determine whether distinct antiviral responses occurred in En-As, En-N, and Uns-C upon TBEV infection, which would reflect differential antiviral responses in human neurons and astrocytes. Cells were infected for 24 h, and the expression of 84 genes of the antiviral response was compared using the same PCR array as previously described. After application of the usual arbitrary cut-off of threefold, 20 genes in TBEV-infected Uns-C, 16 genes in TBEV-infected En-N and 21 genes in TBEV-infected En-As were shown to be significantly upregulated, as compared with their non-infected matched controls (Fig. 7a and Additional file 4). Among the set of upregulated genes, which overlapped with that of non-trypsinized co-cultures, 13 were common to the three cultures (Table 1) while others were specific for En-As or En-N cultures (8/21 and 3/16, respectively). Thus, these results showed that the antiviral program activated by TBEV was partially different in human neurons and astrocytes. Of note, for 12/13 of common genes, the magnitude of upregulation was correlated to the percentage of astrocytes in the cultures. That is, it was much higher in En-As than in En-N and intermediary in Uns-C (Fig. 7a, Table 1), showing that human astrocytes were capable of developing a stronger antiviral response to TBEV than human neurons. To validate the PCR array data and to gain further insight into the kinetics of expression of antiviral genes in each cell types, we performed RT-qPCR at 7, 24, and 72 hpi for IFN-β, (Fig. 7b), 3 PRRs—IFIH1 (MDA5) (Fig. 7c), DDX58 (RIG-I) (Fig. 7d), and TLR3 (Fig. 7e); two ISGs—OAS2 (Fig. 7f) and MX1 (Fig. 7g); and one pro-inflammatory cytokine—CXCL10 (Fig. 7h). Because of their well-known anti-flavivirus activity, the ISGs IFI6 (Fig. 7i), RSAD2 (viperin) (Fig. 7j), and TRIM5α (Fig.7k) were also studied. In confirmation of the PCR array data, all of these genes were significantly more upregulated in En-As than in En-N, at both 24 and 72 hpi. In addition, at 72 hpi, gene expression in En-N was either maintained (RIG-I, TLR3, OAS2, viperin, CXCL10, TRIM5α, IFN-β) or decreased (MDA5, MX1, IFI6), while it was either maintained (MDA5, TLR3, MX1, viperin, TRIM5α, IFN-β) or increased (RIG-I, OAS2, CXCL10, IFI6) in En-As, showing that the duration of antiviral responses was shorter in human neurons than in astrocytes. The induction of an astrocyte-specific antiviral program, as suggested by the selective upregulation of 8 genes in En-As (Fig. 7a, Table 1), was further confirmed by the results of RT-qPCR. Not only TLR3 but also viperin and TRIM5α were among those genes, as upregulation was observed in En-As at 24 hpi and 72 hpi but not in En-N at either time point (Fig. 7e, j, k). Thus, taken together, these results show that TBEV infection induces an antiviral response in human neurons and astrocytes that is characterized by activation of an overlapping set of genes. This antiviral program, however, was of greater intensity and longer duration in human astrocytes than in human neurons. The antiviral programs of the two cell types were also notably distinct, as exemplified by selective upregulation of the genes encoding viperin and TRIM5α, well-known for their anti-TBEV and anti-flavivirus activities [37–39], in human astrocytes. In sum, our results revealed a stronger and broader antiviral response in human astrocytes than in human neurons, in keeping with their differential susceptibility to TBEV infection, astrocytes being more resistant and neurons more susceptible.

**Table 1:** Differential expression of genes involved in the human antiviral response in unsorted cultures (Uns-C), enriched neurons (En-N), and enriched astrocytes (En-As). Up- and downregulated genes appear in bold, after application of a cut-off of 3

Gene name	Fold regulation		
	Uns-C	En-Ne	En-As
CXCL10	**1024.0**	**415.9**	**1209.3**
MX1	**367.1**	**39.1**	**975.5**
CCL5	**680.3**	**265.0**	**814.6**
IFNB1	**149.1**	**71.5**	**537.5**
OAS2	**215.3**	**23.9**	**498.0**
CXCL11	**270.6**	**126.2**	**388.0**
ISG15	**61.8**	**31.8**	**105.4**
IFIH1	**32.7**	**19.7**	**35.3**
DHX58	**13.2**	**10.6**	**28.2**
CASP1	**10.5**	**6.3**	**24.6**
CXCL8	**28.4**	**11.4**	**23.1**
DDX58	**10.5**	**5.7**	**16.0**
IL6	**7.4**	**10.6**	**8.2**
STAT1	**5.8**	2.5	**7.5**
TLR3	**6.2**	1.7	**6.4**
IRF7	**5.0**	0.9	**5.3**
CTSS	**4.3**	1.3	**5.2**
TRIM25	**3.6**	2.2	**4.5**
AIM2	0.9	1.0	**4.0**
MYD88	2.4	1.1	**3.7**
TNF	**4.5**	2.6	**3.7**
IL15	**3.8**	**3.3**	2.3
IFNA1	0.9	**3.6**	1.2
IL12B	0.9	**5.9**	1.2

Differential expression of antiviral genes in human neurons and astrocytes following TBEV infection may reflect differential baseline expression in the two cell types. To test this hypothesis, non-infected En-N, Uns-C, and En-As cells were cultured for 4 days and transcripts from 3 biological samples in each condition were pooled and compared using the human antiviral response PCR array. Antiviral gene expression in En-N was compared with that of Uns-C (Fig. 7l) and En-As (Fig. 7m). Although in both cases, most of the immunity-related genes were not differentially expressed to a significant extent (above the threefold threshold recommended by the manufacturer), we observed that the general tendency was to a slight overexpression in astrocytes, since the number of significantly overexpressed genes increased as the percentage of astrocytes increased in the culture, from Uns-C to En-A. Five/84 genes were, indeed, overexpressed in Uns-C compared with En-N (Fig. 6l), while their number rose to 14/84 genes when En-As were compared with En-N (Fig. 6m). By contrast, very few of these genes (3/84) were overexpressed in human neurons, and then only to a modest extent. To validate these results, differential expression of 3 selected genes, 2 PRRs (MDA5 and RIG-I) and 1 ISG (OAS2), was further addressed by RT-qPCR. Significant overexpression of MDA5 (Fig. 7n) and RIG-I (Fig. 7o) in En-As as compared with En-N was confirmed. By contrast, but still in agreement with the PCR array data, no significant difference was observed for the OAS2 gene (Fig. 7p). These results thus showed that the basal level of expression of certain antiviral genes was higher, albeit slightly, in human astrocytes than in human neurons.

### Human astrocytes protected human neurons from TBEV infection and TBEV-induced damage

**Fig. 8:**
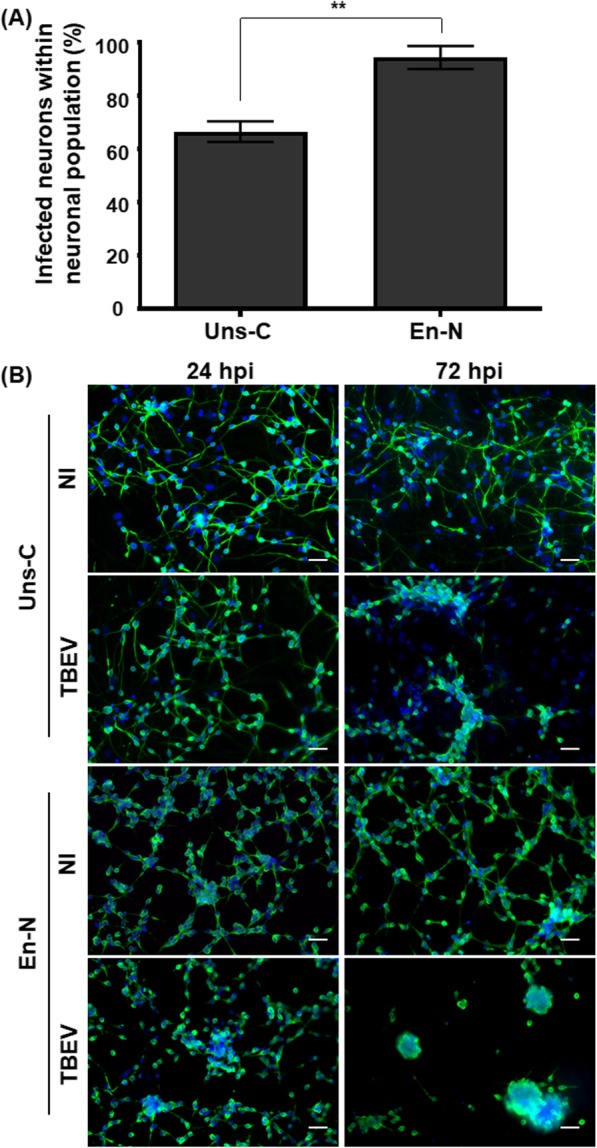
Human astrocytes protect neurons from TBEV infection. Unsorted cells (Uns-C) and enriched neurons (En-N) were infected with TBEV (MOI 10^−2^) and co-immunostained using anti-βIII-tubulin and anti-TBEV-E3 antibodies. **a** Percentage of infected neurons among the neuronal population. Manual enumeration. Data are expressed as the mean ± SD. **b** Immunofluorescence staining of neurons (green). Nuclei were stained with DAPI (blue). Scale bar = 20 μm. Results are representative of two independent experiments performed in triplicate. Statistical analyses were performed with Graphpad Prism V6.0.1 using a two-tailed unpaired *t* test, **p < 0.01

Next, we wondered whether human astrocytes might participate in neuronal defense. We reasoned that, if this were the case, neurons would be more sensitive to TBEV infection in cultures depleted of astrocytes. We therefore compared neuronal susceptibility and vulnerability to TBEV infection in Uns-C and En-N, composed of 20.8 ± 4.9% and 3.1 ± 0.4% of astrocytes, respectively. Uns-C and En-N were infected for 24 h, and the percentage of TBEV-infected neurons within the total neuronal population was quantified based on βIII-tubulin and TBEV-E3 immunostaining. A 30% increase in infected cells was observed in En-N compared with Uns-C (Fig. 8a), showing that, in the absence of astrocytes, neurons were more sensitive to TBEV infection. At that time, neuronal survival was altered neither in TBEV-infected Uns-C nor in TBEV-infected En-N, as revealed by observation of βIII-tubulin immunostaining (Fig. 8b) and enumeration of neurons (see Additional file 5). At 72 hpi, however, a more dramatic alteration of neuronal morphology was observed in TBEV-infected En-N, as compact clusters, characteristic of intense neuronal death, were formed (Fig. 8b). Due to these clusters, it was not possible to enumerate the neurons, but our results clearly showed that, at 72 hpi, neurons were more dramatically affected in cultures deprived of astrocytes. Thus, taken together, these results showed that, upon TBEV infection, the presence of astrocytes was protective for human neurons.

## Discussion

Despite its importance in human health, TBEV-induced neuropathogenesis is still poorly understood. So far, most studies have used either in vitro or in vivo rodent models, whereas these models have advanced understanding, extrapolation to human neuropathogenesis may not always be relevant, as cellular responses display profound differences between species [25, 26, 40]. Here we used neuronal/glial cultures derived from human fetal neural progenitor cells as a more accurate in vitro model to study anti-TBEV innate immunity and its relation to tropism and neuropathogenesis in the human brain. We developed a new in vitro model that mimics major hallmarks of TBEV infection in the human brain, namely neuronal tropism, neuronal death, and astrogliosis, thereby providing a unique and highly relevant pathological model for studying TBEV-induced neuropathogenesis. Moreover, we revealed that a cell type-specific innate antiviral state in human neurons and astrocytes correlates with their differential susceptibility and vulnerability to TBEV, which strongly suggests that the innate antiviral response shapes TBEV tropism for human brain cells.

Understanding viral tropism is critical for understanding virus-induced neuropathogenesis. Some viruses, like Zika virus, preferentially infect neural progenitors [41, 42], whereas human immunodeficiency virus has a strong affinity for microglial cells [43], JC virus for astrocytes [44] and the JHM strain of mouse hepatitis virus (JHMV) for oligodendrocytes [45]. Flaviviruses such as TBEV, WNV, and JEV have a preferential tropism for neurons, a feature that has been observed in human patients [12, 46, 47], as well as in rodent models [48, 49]. Using human neuronal/glial cultures, we reproduced the preferential neuronal tropism that is observed in vivo in showing a high percentage of infected neurons (approximately 55% at the peak of infection) together with a low percentage of infected astrocytes (less than 15%). The limited capacity of the virus to infect astrocytes, as observed in our cultures, as well as in monocultures of rodent or human astrocytes [50–52], may explain the lack of detection of infected astrocytes in post-mortem brain tissues from patients with tick-borne encephalitis [12], or their rare detection in Langat virus-infected mice [49], as infrequently infected cells are likely to escape detection. Similar observations have been made for other neurotropic viruses [53–55]. Unexpectedly, we also observed infection of oligodendrocytes in the human neuronal/glial cultures, a finding that has never been reported previously. The in vivo significance of this observation is at present unknown, especially because the degree of maturation of oligodendrocytes in our culture is undefined, the OLIG2 antibody recognizing mature and immature cells indiscriminately [56]. However, this should be kept in mind for future examination of brain tissues from infected patients. As these cells represent about 5% of the total cell population in our cultures, we believe them to have a minor impact in vitro and, as we could not enrich them, they were not further considered and we confined our study to neurons and astrocytes. We questioned the reasons that may explain the difference in TBEV tropism for these two cell types. This may be due to differential expression of cellular factors that are necessary for establishing a full viral cycle (entry and post-entry events), but the similar percentage of infected neurons and astrocytes that we observed in the first 14 h following TBEV infection does not lend support for this hypothesis. An alternative hypothesis would be differential capacity of the cell types to develop a protective antiviral response. The innate immune response, a major component of the antiviral response, has indeed been proven to be critically important in restricting infection by neurotropic viruses [57–59] and in determining TBEV tropism in different brain areas in murine models [49, 60]. Also, studies using rodent models have shown that distinct brain cell types develop different antiviral states. Microglia and astrocytes, for example, which were initially considered to be the sole sentinels that respond to microbial infection within the brain [61, 62] have been shown to behave differently, as microglia developed a more robust response than astrocytes to TLR7 activation [63]. Neurons, long considered to be merely passive targets, are now known to participate in the antiviral response and viral restriction [19–21, 64], and in humans, neurons and astrocytes have been shown to produce and respond to IFN-α/β. Despite a general assumption that astrocytes are more important players in antiviral response than neurons, the relative contribution of each cell type has, however, not been formally demonstrated, as a direct comparison has never been made, whether in animal or in human in vitro models. Here, we provide the first evidence that human neurons derived from fetal neural progenitor cells possess all of the necessary machinery to mount a cell-intrinsic antiviral response against TBEV, as they upregulated IFN-β, ISGs, and pro-inflammatory cytokine mRNAs upon TBEV infection. We also demonstrated for the first time that human neurons and astrocytes differ in their capacity to mount an anti-TBEV response. Differences were indeed observed in the repertoire of the antiviral program that is activated in the two cell types upon TBEV infection, with certain genes upregulated in astrocytes but not in neurons, such as the RSAD2 (encoding viperin) and TRIM5α genes, two ISGs that have been shown to be highly important for controlling TBEV [37, 38] and other flaviviruses [39, 60] in the rodent’s CNS. Quantitative differences were also observed as transcripts encoding PRRs and genes of IFN signaling were upregulated with different magnitudes in human neurons and astrocytes, with a stronger and more durable response in astrocytes than in neurons. Our results thus show that the cell type-specific anti-TBEV response is correlated with the susceptibility of human neurons and astrocytes to TBEV, which strongly suggests that the innate antiviral response is responsible for shaping TBEV tropism in human brain cells. Such role of innate antiviral response was previously shown for TBEV and other flaviviruses in different neuronal sub-types of murine origin [19, 60]. Whether the high neuronal susceptibility to TBEV infection in the human neuronal/glial cultures is due to a weaker general antiviral program in human neurons, involving multiple components of the IFN response, from the PRRs to ISGs, or rather to lower expression of specific ISGs, such as the RSAD2, IFI6, and TRIM5α genes, that are dedicated to the control of flaviviruses [30], remains to be elucidated. It also remains to be determined whether the antiviral response of neurons is weak in response to infection by any neurotropic virus, or only by certain viruses, such as flaviviruses, or whether it is specific to TBEV infection. Our observation that the baseline expression of certain antiviral genes is lower in neurons than in astrocytes may argue in favor of the first possibility, a hypothesis that should be addressed in future studies. In contrast to our results, TBEV infection in the human neuronal DAOY cells, a human neuroblastoma, led to upregulation of the RSAD2 gene [65], a discrepancy that may be due to the use of non-physiological, immortalized cells in the study in question. Of note, our results showed that neuronal tropism of TBEV does not depend only on the cell-specific antiviral response in human neurons, as the presence of astrocytes in the culture limited their infection and favored their survival. As it was previously reported that murine astrocytes infected with TBEV were protective to neurons through IFN signaling [50], and as we showed that IFNβ was highly upregulated by human astrocytes upon TBEV infection, we speculate that IFNβ produced by astrocytes acts in a paracrine manner to restrict neuronal infection in our human neuronal/glial co-cultures. However, it cannot be ruled out that other yet unknown factors may be involved.

Viral tropism and pathogenesis are intimately linked, but how the former governs the latter in the human CNS, during TBEV infection, is incompletely understood. TBEV preferentially infects and kills the neurons [12], a highly dramatic event, as neurons have a very poor capacity to regenerate. Neuronal death may occur in either direct or indirect manners, such as, in the latter case, by inducing secretion of neurotoxic proteins by resident glial cells or recruitment of peripheral inflammatory cells to the brain parenchyma [11, 14]. Involvement of T cells in neuronal death cannot be explored in our model. However, it should be noted that chemokines such as CXCL10, CCL5, and CXCL11, which have been shown to be overexpressed in the cerebrospinal fluid of human patients infected with TBEV [66–68], are highly upregulated by both human neurons and astrocytes, revealing that the two cell types may participate in chemo-attraction of T cells into TBEV-infected human brain parenchyma. Similar to the observation made for PRRs and ISGs, their upregulation was, however, stronger in astrocytes than in neurons, showing that astrocytes may be a major player in this process as well. Regarding neuronal death due to direct infection by TBEV, it has not yet been demonstrated, although ultrastructural changes in response to TBEV infection have been observed [69, 70]. In our human neuronal/glial and enriched neuron cultures, neuronal death occurred in the absence of peripheral cells and was associated with infection of a large proportion of neurons, showing that the virus is directly responsible for their death. This is likely to play an important role in the human brain upon infection with TBEV and most probably other flaviviruses, since similar conclusions have been drawn for West Nile virus in studies performed in primary murine neurons [71]. Reactive astrocytes, however, may also influence neuronal death. Astrogliosis, indeed, occurs following brain trauma of diverse etiology [72], including infection by TBEV or the related Langat virus infection [12], and may be either beneficial or detrimental to neurons [73]. In human neuronal/glial cultures, we have observed that astrocytes were hypertrophic, a classical feature of reactive astrocytes and, when neurons were deprived of astrocytes, they were more sensitive to TBEV infection, showing that astrocytes exerted, under these conditions, a neuroprotective effect, possibly via restriction of neuronal infection as previously discussed. Thus, the intensity of neuronal death depends not only on direct infection of neurons but also on the indirect effect mediated by reactive astrocytes. Proliferation sometimes accompanied astrocyte hypertrophy in astrogliosis. Surprisingly, in our experiments, we observed a decrease in the number of astrocytes by 7 days following infection, which strongly suggested that TBEV induced astrocytic death. This is in apparent contradiction with previous work showing that astrocyte viability was unaffected in rodent and human astrocyte monocultures [51, 52]. The difference in our results may be due to differential conditions of infection, such as viral strain or infectious dose or to the presence of neurons in our co-cultures, which may lead to astrocyte death by an unknown mechanism. Of note, infection with TBEV is not always correlated to cell death, since as many as 60% of oligodendrocytes were infected without impact on their viability. Why certain cell types die upon TBEV infection while others do not remains to be understood, as do the molecular pathways that lead to cell death. Interestingly, our findings suggest that neuronal death may result from axonal pathology and retrograde degeneration. Indeed, we showed that loss of neurites preceded the disappearance of neuronal cell bodies, an observation that is in agreement with previous work in which accumulation of viral protein in neuritic extensions and dendritic degeneration due to local replication of TBEV was evidenced in murine neurons [69]. The relative contribution of axonal degeneration, which is known to play a pathogenic role in rabies virus infection [74] and in some neurodegenerative diseases [75], to TBEV-induced neuronal death need to be defined in future studies.

## Conclusions

**Fig. 9:**
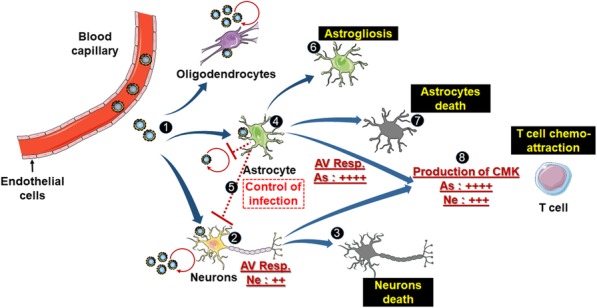
Proposed model of interactions between TBEV and human brain cells. In the human brain parenchyma, TBEV infects neurons, astrocytes, and possibly oligodendrocytes (1). Both neurons and astrocytes develop an antiviral response. In neurons, it is insufficient to afford protection (2) and poorly controlled infection induces neuronal death in a direct manner (3). Astrocytes are infected but control infection, owing to their strong antiviral response (4), which may also be beneficial to neurons (5). Astrocytes enter a reactive stage (6) and some of them die (7). Both neurons and astrocytes overexpressed a high level of chemokines involved in chemo-attraction of T cells in the brain parenchyma (8), although astrocytes are stronger producers. The figure was created using Servier Medical Art available on www.servier.com. As, astrocytes; AV Resp, antiviral response; CMK, chemokines; Ne, neurons

Until recently, the lack of relevant in vitro models virtually precluded meaningful study of viral pathogenesis in the human brain. This obstacle has been overcome by the development of methodologies providing an unlimited source of human neural cell types that can be used for disease modeling. In this study, we set up a new, complex, and highly relevant in vitro model that mimics the major events of TBEV infection in the human brain. Using this model, we evidenced differential innate immune responses in human neurons and astrocytes that contribute to shaping TBEV tropism and neuropathogenesis. Based on our results, we propose a model for interactions between TBEV and human brain cells that is represented in Fig. 9. Our study thus advances understanding of the mechanisms involved in TBEV-induced damage of the human brain and provides a pathological model that can be used in the future to provide greater knowledge as well as to develop new therapies by screening for antiviral or neuroprotective drugs.

## Supplementary information

**Additional file 1.** Primer pairs used for qRT-PCR analyses.

**Additional file 2.** Neurons and astrocytes are the major cell types in hNPCs-derived cultures. HNPCs were differentiated for 13 days. (A) Immunofluorescence labeling using antibodies against HuC/HuD, a neuronal nuclear marker (green), GFAP, an astrocytic marker (red) and OLIG2, an oligodendrocyte nuclear marker (gray) were used. Nuclei were stained with DAPI (blue). Scale bar = 20 μm. (B) Enumeration of cells based on immunofluorescence labeling. Automated quantification using an ArrayScan Cellomics instrument. (C) Enumeration of astrocytes based on immunofluorescence labeling. Manual quantification.

**Additional file 3.** TBEV-induced antiviral response in neuronal/glial cells (PCR array data, 24 hpi).

**Additional file 4.** Antiviral response in enriched neurons, astrocytes and unsorted cells (PCR array data, 24 hpi).

**Additional file 5 **Neuronal survival is not affected by TBEV infection at 24 hpi in unsorted cells and enriched neuron cultures. Unsorted cultures (Uns-C) and enriched neurons (En-N) were infected with TBEV and co-immunostained with βIII-tubulin (neurons) and anti-TBEV-E3 antibodies. Manual enumeration of infected neurons was performed at 24hpi. Data are expressed as the mean ± SD and normalized to non-infected Uns-C. Results are representative of two independent experiments performed in triplicate. Statistical analysis was performed using a two-tailed unpaired t test with GraphPad Prism V6.0.1, ns = non-significant (*p* > 0.05).

## Data Availability

Data supporting the conclusions of this work have been presented in the manuscript.

## Abbreviations

BSL-3: Bio-safety level-3
CNS: Central nervous system
Co-C: Non-trypsinized co-cultures
d13/d21: Day 13 or 21 after the onset of differentiation
DAPI: 4′,6-Diamidino-2-phenylindole
DCs: Dendritic cells
En-As: Enriched astrocytes
En-N: Enriched neurons
hESCs: Human embryonic stem cells
hiPSCs: Human induced pluripotent stem cells
hNPCs: Human neural progenitor cells
hpi: Hours post-infection
IFN: Interferon
IFNAR: IFN alpha/beta receptor
ISG: Interferon-stimulated genes
JEV: Japanese encephalitis virus
JHMV: JHM strain of mouse hepatitis virus
POWV: Powassan virus
PRRs: Pathogen recognition receptors
TBEV: Tick-borne encephalitis virus
Uns-C: Unsorted sub-cultured cells
WNV: West Nile virus
ZIKV: Zika virus
