# Supplementary material for: Pathological modeling of TBEV infection reveals differential innate immune responses in human neurons and astrocytes that correlate with their susceptibility to infection
Source: J Neuroinflammation. 2020 Mar 3;17:76. doi: 10.1186/s12974-020-01756-x (PMC7053149; doi:10.1186/s12974-020-01756-x)
Supplement: Supplementary file 1 — Additional file 1. Primer pairs used for qRT-PCR analyses. [file 12974_2020_1756_MOESM1_ESM.docx]

**Additional file 1 (table.doc). Primer pairs used for qRT-PCR analyses.**

| **Gene name** | **3’ primer** | **5’ primer** |
| --- | --- | --- |
| GAPDH | CACCATCTTCCAGGAGCGAG | GAGAtgAtgACCCTTTTGGC |
| HPRT1 | GGACTAATTAtgGACAGGACTG | GCTCTTCAGTCTGATAAAATCTAC |
| TBEV | GGGCGGTTCTTGTTCTCC | ACACATCACCTCCTTGTCAGACT |
| TLR3 | GCTGCAGTCAGCAACTTCAT | AGGAAAGGCTAGCAGTCATCC |
| DDX58 (RIG-I) | GAGAAAAAGTGTGGCAGCCT | ATATCCGGAAGACCCTGGAC |
| IFIH1 (MDA5) | TGCCCAtgTTGCTGTTAtgT | GTCTGGGGCAtgGAGAATAA |
| CXCL10 | GCAGGTACAGCGTACGGTTC | CAGCAGAGGAACCTCCAGTC |
| CXCL11 | AtgCAAAGACAGCGTCCTCT | CAAACAtgAGTGTGAAGGGC |
| CCL5 (RANTES) | TGTACTCCCGAACCCATTTC | TACACCAGTGGCAAGTGCTC |
| RSAD2 (viperin) | GTCCCTGGCATACAGAGACTG | GCTCAGAGGTTGCCTGAACA |
| IFI6 | TCGCTGAtgAGCTGGTCTGC | ATTACCTAtgACGACGCTGC |
| OAS2 | TGTTTTCCGTCCATAGGAGC | CTGATCGACGAGAtgGTGAA |
| MX1 | CTACACACCGTGACGGATAtg | CGAGCTGGATTGGAAAGCCC |
| ISG15 | CACCGTGTTCAtgAATCTGC | CTTTATTTCCGGCCCTTGAT |
| IFN-β | CAACTTGCTTGGATTCCTACAAAG | TATTCAAGCCTCCCATTCAATTG |
| TRIM5α | TGCCTCTGACACTGACTAAGAAGAtg | GGGCTAAGGACTCATTCATTGG |
