# Supplementary figures and images for: Pathological modeling of TBEV infection reveals differential innate immune responses in human neurons and astrocytes that correlate with their susceptibility to infection
Source: J Neuroinflammation. 2020 Mar 3;17:76. doi: 10.1186/s12974-020-01756-x (PMC7053149; doi:10.1186/s12974-020-01756-x)

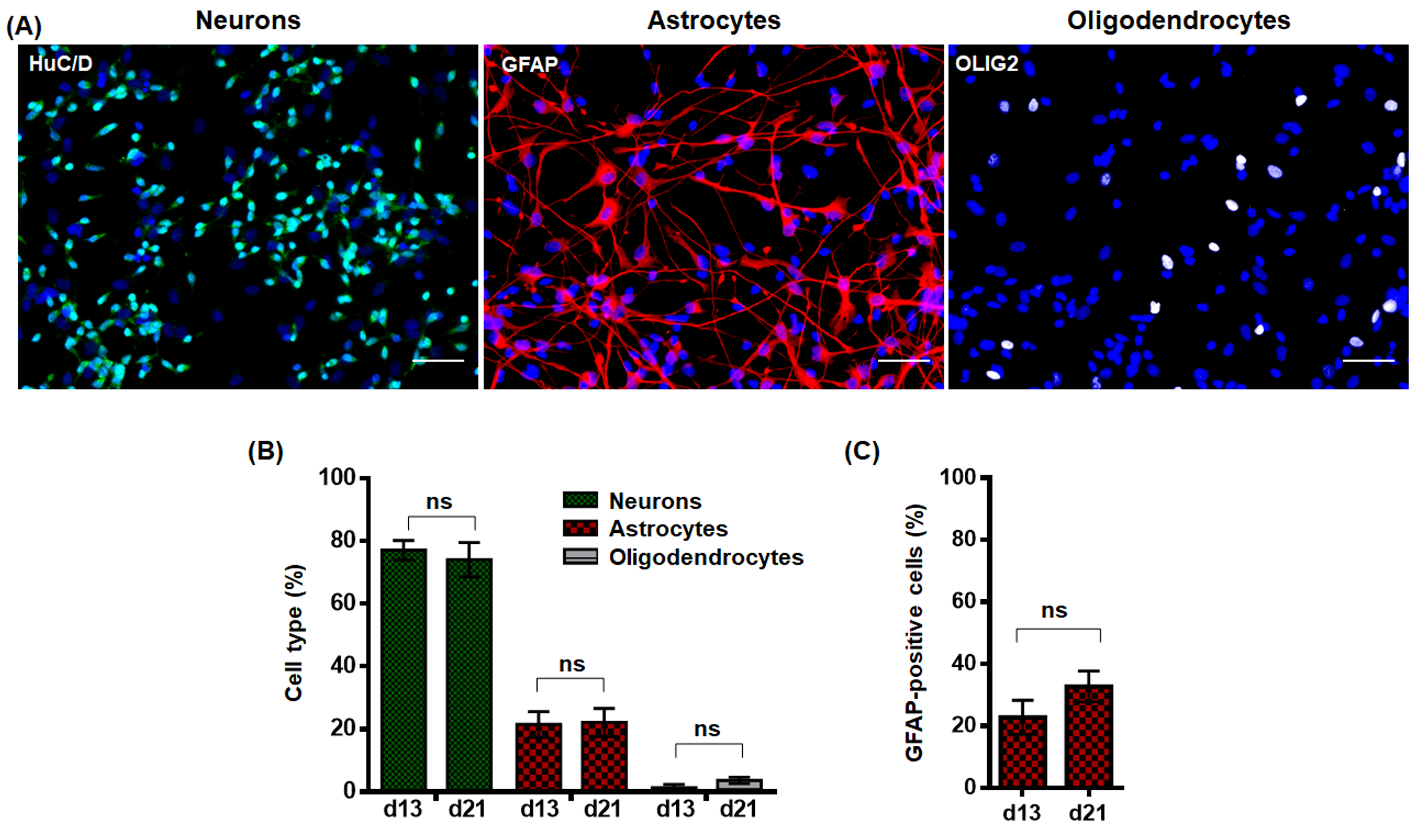

Supplement: Supplementary file 2 — Additional file 2. Neurons and astrocytes are the major cell types in hNPCs-derived cultures. HNPCs were differentiated for 13 days. (A) Immunofluorescence labeling using antibodies against HuC/HuD, a neuronal nuclear marker (green), GFAP, an astrocytic marker (red) and OLIG2, an oligodendrocyte nuclear marker (gray) were used. Nuclei were stained with DAPI (blue). Scale bar = 20 μm. (B) Enumeration of cells based on immunofluorescence labeling. Automated quantification using an ArrayScan Cellomics instrument. (C) Enumeration of astrocytes based on immunofluorescence labeling. Manual quantification. [file 12974_2020_1756_MOESM2_ESM.tif]

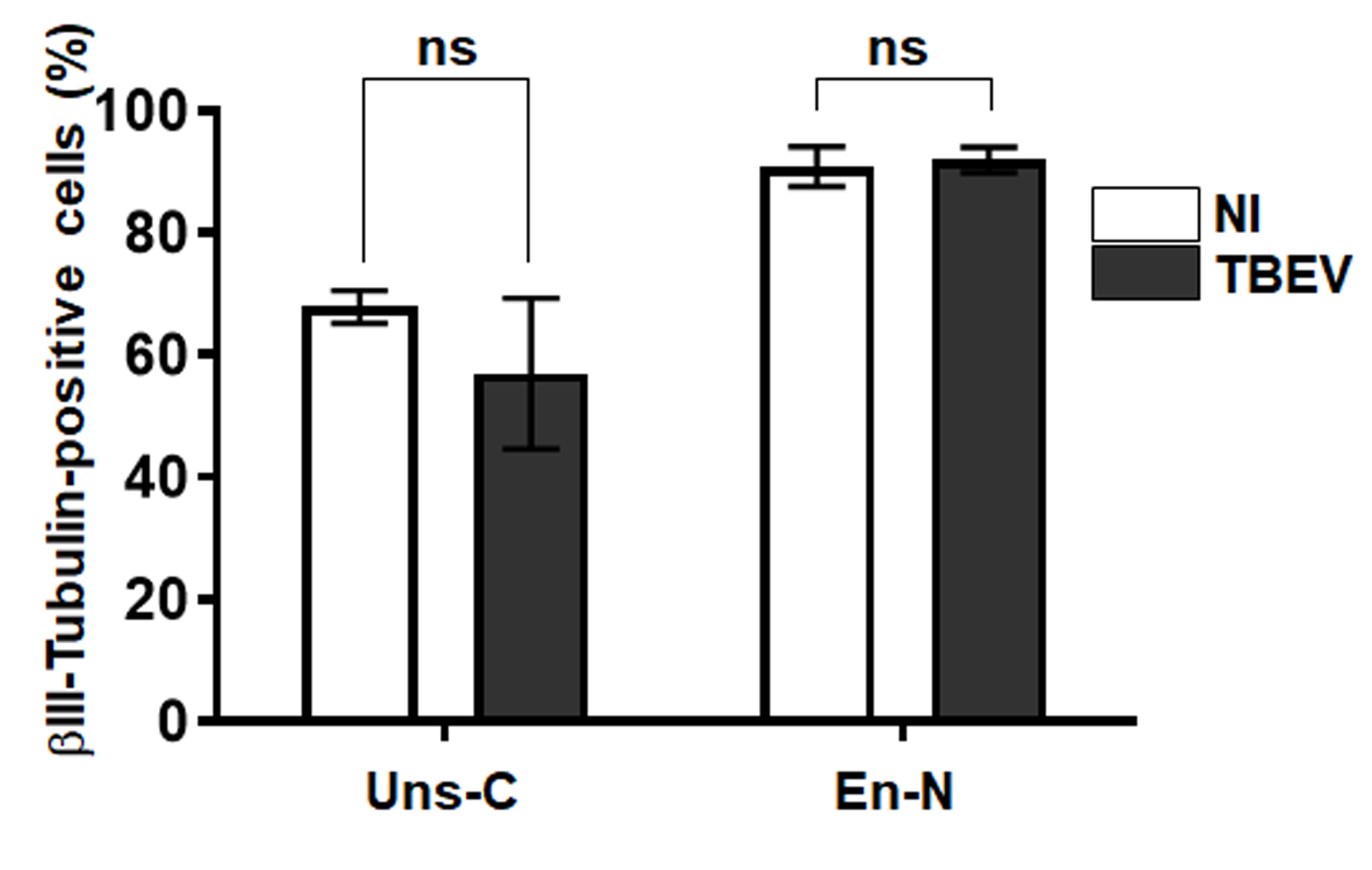

Supplement: Supplementary file 5 — Additional file 5 Neuronal survival is not affected by TBEV infection at 24 hpi in unsorted cells and enriched neuron cultures. Unsorted cultures (Uns-C) and enriched neurons (En-N) were infected with TBEV and co-immunostained with βIII-tubulin (neurons) and anti-TBEV-E3 antibodies. Manual enumeration of infected neurons was performed at 24hpi. Data are expressed as the mean ± SD and normalized to non-infected Uns-C. Results are representative of two independent experiments performed in triplicate. Statistical analysis was performed using a two-tailed unpaired t test with GraphPad Prism V6.0.1, ns = non-significant (p > 0.05). [file 12974_2020_1756_MOESM5_ESM.tif]
