# Supplementary material for: Pathological modeling of TBEV infection reveals differential innate immune responses in human neurons and astrocytes that correlate with their susceptibility to infection
Source: J Neuroinflammation. 2020 Mar 3;17:76. doi: 10.1186/s12974-020-01756-x (PMC7053149; doi:10.1186/s12974-020-01756-x)
Supplement: Supplementary file 3 — Additional file 3. TBEV-induced antiviral response in neuronal/glial cells (PCR array data, 24 hpi). [file 12974_2020_1756_MOESM3_ESM.docx]

**Additional file 3 (table.doc). TBEV-induced antiviral response in neuronal/glial cells (PCR array data, 24 hpi)**

| Gene symbol | Ct | | Fold Regulation |  | Gene symbol | Ct | | Fold Regulation |
| --- | --- | --- | --- | --- | --- | --- | --- | --- |
|  | NI | TBEV |  |  |  | NI | TBEV |  |
| AIM2 | 32.97 | 33.03 | 1.68 |  | IRF3 | 27.94 | 27.66 | 1.15 |
| APOBEC3G | 39.34 | 34.20 | 6.41 |  | IRF5 | 32.90 | 32.90 | -1.09 |
| Atg5 | 26.79 | 27.04 | 1.09 |  | IRF7 | 31.06 | 28.63 | 5.03 |
| AZI2 | 26.39 | 26.37 | 1.69 |  | ISG15 | 30.05 | 23.98 | 203.66 |
| CARD9 | n.d. | 33.86 | -1.30 |  | JUN | 28.24 | 27.72 | 1.62 |
| CASP1 | 31.21 | 27.70 | 37.01 |  | MAP2K1 | 27.85 | 27.84 | 1.10 |
| CASP10 | 33.23 | 34.57 | -3.16 |  | MAP2K3 | 29.16 | 28.59 | 1.46 |
| CASP8 | 31.37 | 31.28 | -1.21 |  | MAP3K1 | 27.95 | 28.19 | 1.32 |
| CCL3 | 41.57 | 36.03 | 1.04 |  | MAP3K7 | 26.58 | 26.90 | 1.30 |
| CCL5 | 43.49 | 25.47 | 207.94 |  | MAPK1 | 24.96 | 25.16 | 1.23 |
| CD40 | 43.95 | n.d. | -3.89 |  | MAPK14 | 27.69 | 28.00 | 1.04 |
| CD80 | 35.43 | 41.75 | 1.13 |  | MAPK3 | 28.12 | 28.31 | -1.04 |
| CD86 | n.d. | n.d. | 2.97 |  | MAPK8 | 26.01 | 26.65 | 1.19 |
| CHUK | 28.51 | 28.60 | 1.31 |  | MAVS | 28.34 | 28.59 | -1.03 |
| CTSB | 26.48 | 26.85 | 1.24 |  | MEFV | 33.87 | 35.09 | 3.18 |
| CTSL | 26.49 | 27.13 | 1.09 |  | MX1 | 32.55 | 23.91 | 522.76 |
| CTSS | 33.84 | 31.63 | 3.27 |  | MYD88 | 29.03 | 27.64 | 3.14 |
| CXCL10 | 43.64 | 24.88 | 719.08 |  | NFKB1 | 30.59 | 29.38 | 2.28 |
| CXCL11 | n.d. | 26.80 | 188.71 |  | NFKBIA | 28.71 | 27.45 | 3.03 |
| CXCL9 | 30.76 | 29.77 | 5.70 |  | NLRP3 | n.d. | n.d. | -4.06 |
| CYLD | 28.93 | 28.14 | 1.72 |  | NOD2 | 41.52 | 35.41 | 2.41 |
| TKFC | 30.55 | 30.51 | -1.16 |  | OAS2 | 42.50 | 27.13 | 541.19 |
| DDX3X | 25.08 | 25.36 | 1.13 |  | PIN1 | 27.56 | 27.80 | 1.04 |
| DDX58 | 27.86 | 24.35 | 17.63 |  | PSTPIP1 | 32.96 | 32.43 | -1.48 |
| DHX58 | 35.28 | 31.16 | 99.04 |  | PYCARD | 33.06 | 33.49 | -1.05 |
| FADD | 28.93 | 29.37 | 1.46 |  | PYDC1 | 34.99 | 36.14 | 2.19 |
| FOS | 27.24 | 26.86 | 1.67 |  | RELA | 27.67 | 27.46 | 1.31 |
| HSP90AA1 | 23.08 | 23.12 | 1.25 |  | RIPK1 | 29.37 | 28.72 | 2.20 |
| IFIH1 | 29.90 | 24.75 | 42.81 |  | SPP1 | 30.22 | 29.52 | 1.15 |
| IFNA1 | n.d. | 35.11 | -1.68 |  | STAT1 | 26.78 | 24.13 | 11.63 |
| IFNA2 | n.d. | n.d. | 1.30 |  | SUGT1 | 25.57 | 25.93 | 1.36 |
| IFNAR1 | 25.69 | 26.18 | -1.09 |  | TBK1 | 28.40 | 28.26 | 1.35 |
| IFNB1 | 33.74 | 26.40 | 44.32 |  | TICAM1 | 30.78 | 29.84 | 1.46 |
| IKBKB | 27.96 | 28.02 | 1.20 |  | TLR3 | 30.61 | 27.85 | 8.40 |
| IL12A | 30.19 | 30.55 | 1.78 |  | TLR7 | 37.65 | 37.79 | 1.04 |
| IL12B | 35.59 | 35.01 | 2.19 |  | TLR8 | n.d. | n.d. | 1.04 |
| IL15 | 34.82 | 32.78 | 6.45 |  | TLR9 | 34.15 | 42.40 | -1.04 |
| IL18 | 33.00 | 34.45 | 1.17 |  | TNF | 40.18 | 32.70 | -1.16 |
| IL1B | 39.11 | 34.31 | -2.07 |  | TRADD | 33.63 | 33.08 | 1.59 |
| IL6 | 34.41 | 31.40 | -1.10 |  | TRAF3 | 29.00 | 28.98 | 1.04 |
| CXCL8 | 37.46 | 30.05 | 12.55 |  | TRAF6 | 27.70 | 27.47 | 1.14 |
| IRAK1 | 29.70 | 29.54 | 1.29 |  | TRIM25 | 27.49 | 25.52 | 6.41 |
