# Supplementary material for: Pathological modeling of TBEV infection reveals differential innate immune responses in human neurons and astrocytes that correlate with their susceptibility to infection
Source: J Neuroinflammation. 2020 Mar 3;17:76. doi: 10.1186/s12974-020-01756-x (PMC7053149; doi:10.1186/s12974-020-01756-x)
Supplement: Supplementary file 4 — Additional file 4. Antiviral response in enriched neurons, astrocytes and unsorted cells (PCR array data, 24 hpi). [file 12974_2020_1756_MOESM4_ESM.docx]

**Additional file 4 (table.doc). Antiviral response in enriched neurons, astrocytes and unsorted cells (PCR array data, 24 hpi)**

| **Unsorted cultures** | | | | | | | | | | | |
| --- | --- | --- | --- | --- | --- | --- | --- | --- | --- | --- | --- |
| Gene symbol | Ct | | Fold Regulation |  | Gene symbol | Ct | | | Fold Regulation | |  |
|  | NI | TBEV |  |  |  | NI | TBEV |  | |  |  |
| AIM2 | 32.97 | 33.03 | -1.13 |  | IRF3 | 27.94 | 27.66 | 1.12 | |  |  |
| APOBEC3G | 35.00 | 34.20 | 1.60 |  | IRF5 | 32.90 | 32.90 | -1.09 | |  |  |
| ATG5 | 26.79 | 27.04 | -1.29 |  | IRF7 | 31.06 | 28.63 | 4.96 | |  |  |
| AZI2 | 26.39 | 26.37 | -1.07 |  | ISG15 | 30.05 | 23.98 | 61.82 | |  |  |
| CARD9 | 35.00 | 33.86 | 2.03 |  | JUN | 28.24 | 27.72 | 1.32 | |  |  |
| CASP1 | 31.21 | 27.70 | 10.48 |  | MAP2K1 | 27.85 | 27.84 | -1.08 | |  |  |
| CASP10 | 33.23 | 34.57 | -2.75 |  | MAP2K3 | 29.16 | 28.59 | 1.37 | |  |  |
| CASP8 | 31.37 | 31.28 | -1.02 |  | MAP3K1 | 27.95 | 28.19 | -1.28 | |  |  |
| CCL3 | 35.00 | 35.00 | -1.09 |  | MAP3K7 | 26.58 | 26.90 | -1.36 | |  |  |
| CCL5 | 35.00 | 25.47 | 680.29 |  | MAPK1 | 24.96 | 25.16 | -1.25 | |  |  |
| CD40 | 35.00 | 35.00 | -1.09 |  | MAPK14 | 27.69 | 28.00 | -1.35 | |  |  |
| CD80 | 35.00 | 35.00 | -1.09 |  | MAPK3 | 28.12 | 28.31 | -1.24 | |  |  |
| CD86 | 35.00 | 35.00 | -1.09 |  | MAPK8 | 26.01 | 26.65 | -1.69 | |  |  |
| CHUK | 28.51 | 28.60 | -1.16 |  | MAVS | 28.34 | 28.59 | -1.29 | |  |  |
| CTSB | 26.48 | 26.85 | -1.40 |  | MEFV | 33.87 | 35.00 | -2.38 | |  |  |
| CTSL | 26.49 | 27.13 | -1.69 |  | MX1 | 32.55 | 23.91 | 367.09 | |  |  |
| CTSS | 33.84 | 31.63 | 4.26 |  | MYD88 | 29.03 | 27.64 | 2.41 | |  |  |
| CXCL10 | 35.00 | 24.88 | 1024.00 |  | NFKB1 | 30.59 | 29.38 | 2.13 | |  |  |
| CXCL11 | 35.00 | 26.80 | 270.60 |  | NFKBIA | 28.71 | 27.45 | 2.20 | |  |  |
| CXCL9 | 30.76 | 29.77 | 1.83 |  | NLRP3 | 35.00 | 35.00 | -1.09 | |  |  |
| CYLD | 28.93 | 28.14 | 1.59 |  | NOD2 | 35.00 | 35.00 | -1.09 | |  |  |
| TKFC | 30.55 | 30.51 | -1.06 |  | OAS2 | 35.00 | 27.13 | 215.27 | |  |  |
| DDX3X | 25.08 | 25.36 | -1.32 |  | PIN1 | 27.56 | 27.80 | -1.28 | |  |  |
| DDX58 | 27.86 | 24.35 | 10.48 |  | PSTPIP1 | 32.96 | 32.43 | 1.33 | |  |  |
| DHX58 | 35.00 | 31.16 | 13.18 |  | PYCARD | 33.06 | 33.49 | -1.46 | |  |  |
| FADD | 28.93 | 29.37 | -1.47 |  | PYDC1 | 34.99 | 35.00 | -1.09 | |  |  |
| FOS | 27.24 | 26.86 | 1.20 |  | RELA | 27.67 | 27.46 | 1.06 | |  |  |
| HSP90AA1 | 23.08 | 23.12 | -1.12 |  | RIPK1 | 29.37 | 28.72 | 1.44 | |  |  |
| IFIH1 | 29.90 | 24.75 | 32.67 |  | SPP1 | 30.22 | 29.52 | 1.49 | |  |  |
| IFNA1 | 35.00 | 35.00 | -1.09 |  | STAT1 | 26.78 | 24.13 | 5.78 | |  |  |
| IFNA2 | 35.00 | 35.00 | -1.09 |  | SUGT1 | 25.57 | 25.93 | -1.39 | |  |  |
| IFNAR1 | 25.69 | 26.18 | -1.53 |  | TBK1 | 28.40 | 28.26 | 1.01 | |  |  |
| IFNB1 | 33.74 | 26.40 | 149.09 |  | TICAM1 | 30.78 | 29.84 | 1.77 | |  |  |
| IKBKB | 27.96 | 28.02 | -1.13 |  | TLR3 | 30.61 | 27.85 | 6.23 | |  |  |
| IL12A | 30.19 | 30.55 | -1.39 |  | TLR7 | 35.00 | 35.00 | -1.09 | |  |  |
| IL12B | 35.00 | 35.00 | -1.09 |  | TLR8 | 35.00 | 35.00 | -1.09 | |  |  |
| IL15 | 34.82 | 32.78 | 3.78 |  | TLR9 | 34.15 | 35.00 | -1.96 | |  |  |
| IL18 | 33.00 | 34.45 | -2.97 |  | TNF | 35.00 | 32.70 | 4.53 | |  |  |
| IL1B | 35.00 | 34.31 | 1.48 |  | TRADD | 33.63 | 33.08 | 1.35 | |  |  |
| IL6 | 34.41 | 31.40 | 7.41 |  | TRAF3 | 29.00 | 28.98 | -1.07 | |  |  |
| CXCL8 | 35.00 | 30.05 | 28.44 |  | TRAF6 | 27.70 | 27.47 | 1.08 | |  |  |
| IRAK1 | 29.70 | 29.54 | 1.03 |  | TRIM25 | 27.49 | 25.52 | 3.61 | |  |  |

| **En-Neurons** | | | | | | | | | | | | | | | | | |  |  |
| --- | --- | --- | --- | --- | --- | --- | --- | --- | --- | --- | --- | --- | --- | --- | --- | --- | --- | --- | --- |
| Gene symbol | Ct | | | | Fold Regulation | |  | | Gene symbol | | Ct | | | | Fold Regulation | |  |  |  |
|  | NI | | TBEV | |  |  |  | |  |  | NI | | TBEV | |  |  |  |  |  |
| AIM2 | 32.73 | | 33.05 | | 1.01 | |  | | IRF3 | | 28.93 | | 29.16 | | 1.08 | |  |  |  |
| APOBEC3G | 35.00 | | 35.00 | | 1.27 | |  | | IRF5 | | 32.93 | | 33.52 | | -1.19 | |  |  |  |
| ATG5 | 26.97 | | 27.56 | | -1.19 | |  | | IRF7 | | 30.81 | | 31.27 | | -1.09 | |  |  |  |
| AZI2 | 26.50 | | 26.61 | | 1.17 | |  | | ISG15 | | 30.99 | | 26.34 | | 31.78 | |  |  |  |
| CARD9 | 32.71 | | 33.51 | | -1.38 | |  | | JUN | | 28.87 | | 29.17 | | 1.03 | |  |  |  |
| CASP1 | 33.39 | | 31.08 | | 6.28 | |  | | MAP2K1 | | 28.39 | | 28.95 | | -1.16 | |  |  |  |
| CASP10 | 32.98 | | 33.60 | | -1.21 | |  | | MAP2K3 | | 30.14 | | 30.01 | | 1.39 | |  |  |  |
| CASP8 | 31.62 | | 32.51 | | -1.46 | |  | | MAP3K1 | | 28.17 | | 28.74 | | -1.17 | |  |  |  |
| CCL3 | 35.00 | | 34.45 | | 1.85 | |  | | MAP3K7 | | 26.72 | | 26.83 | | 1.17 | |  |  |  |
| CCL5 | 33.93 | | 26.22 | | 265.03 | |  | | MAPK1 | | 25.73 | | 26.22 | | -1.11 | |  |  |  |
| CD40 | 35.00 | | 35.00 | | 1.27 | |  | | MAPK14 | | 27.52 | | 28.17 | | -1.24 | |  |  |  |
| CD80 | 33.96 | | 35.00 | | -1.62 | |  | | MAPK3 | | 28.50 | | 29.10 | | -1.20 | |  |  |  |
| CD86 | 35.00 | | 35.00 | | 1.27 | |  | | MAPK8 | | 25.39 | | 26.01 | | -1.21 | |  |  |  |
| CHUK | 28.34 | | 28.72 | | -1.03 | |  | | MAVS | | 28.72 | | 29.50 | | -1.36 | |  |  |  |
| CTSB | 26.95 | | 27.60 | | -1.24 | |  | | MEFV | | 35.00 | | 35.00 | | 1.27 | |  |  |  |
| CTSL | 27.77 | | 28.08 | | 1.02 | |  | | MX1 | | 32.36 | | 27.41 | | 39.12 | |  |  |  |
| CTSS | 32.86 | | 32.85 | | 1.27 | |  | | MYD88 | | 29.36 | | 29.61 | | 1.06 | |  |  |  |
| CXCL10 | 35.00 | | 26.64 | | 415.87 | |  | | NFKB1 | | 30.97 | | 30.54 | | 1.71 | |  |  |  |
| CXCL11 | 35.00 | | 28.36 | | 126.24 | |  | | NFKBIA | | 28.41 | | 28.05 | | 1.62 | |  |  |  |
| CXCL9 | 30.55 | | 30.17 | | 1.65 | |  | | NLRP3 | | 35.00 | | 35.00 | | 1.27 | |  |  |  |
| CYLD | 27.96 | | 28.23 | | 1.05 | |  | | NOD2 | | 35.00 | | 35.00 | | 1.27 | |  |  |  |
| TKFC | 30.68 | | 31.61 | | -1.51 | |  | | OAS2 | | 34.03 | | 29.79 | | 23.92 | |  |  |  |
| DDX3X | 25.63 | | 26.04 | | -1.05 | |  | | PIN1 | | 27.86 | | 28.63 | | -1.35 | |  |  |  |
| DDX58 | 28.61 | | 26.43 | | 5.74 | |  | | PSTPIP1 | | 32.73 | | 33.50 | | -1.35 | |  |  |  |
| DHX58 | 35.00 | | 31.94 | | 10.56 | |  | | PYCARD | | 31.60 | | 32.44 | | -1.41 | |  |  |  |
| FADD | 29.19 | | 30.17 | | -1.56 | |  | | PYDC1 | | 35.00 | | 35.00 | | 1.27 | |  |  |  |
| FOS | 28.88 | | 29.03 | | 1.14 | |  | | RELA | | 28.09 | | 28.47 | | -1.03 | |  |  |  |
| HSP90AA1 | 23.72 | | 24.35 | | -1.22 | |  | | RIPK1 | | 30.08 | | 29.92 | | 1.41 | |  |  |  |
| IFIH1 | 31.25 | | 27.29 | | 19.70 | |  | | SPP1 | | 30.60 | | 31.39 | | -1.37 | |  |  |  |
| IFNA1 | 35.00 | | 33.48 | | 3.63 | |  | | STAT1 | | 28.01 | | 27.02 | | 2.51 | |  |  |  |
| IFNA2 | 35.00 | | 35.00 | | 1.27 | |  | | SUGT1 | | 25.52 | | 25.94 | | -1.06 | |  |  |  |
| IFNAR1 | 26.36 | | 27.10 | | -1.32 | |  | | TBK1 | | 28.77 | | 28.85 | | 1.20 | |  |  |  |
| IFNB1 | 32.91 | | 27.09 | | 71.51 | |  | | TICAM1 | | 31.80 | | 31.58 | | 1.47 | |  |  |  |
| IKBKB | 28.51 | | 29.15 | | -1.23 | |  | | TLR3 | | 31.35 | | 30.92 | | 1.71 | |  |  |  |
| IL12A | 32.92 | | 32.43 | | 1.78 | |  | | TLR7 | | 35.00 | | 35.00 | | 1.27 | |  |  |  |
| IL12B | 35.00 | | 32.77 | | 5.94 | |  | | TLR8 | | 34.89 | | 35.00 | | 1.17 | |  |  |  |
| IL15 | 34.97 | | 33.60 | | 3.27 | |  | | TLR9 | | 32.28 | | 33.48 | | -1.82 | |  |  |  |
| IL18 | 31.39 | | 32.42 | | -1.61 | |  | | TNF | | 35.00 | | 33.96 | | 2.60 | |  |  |  |
| IL1B | 35.00 | | 34.98 | | 1.28 | |  | | TRADD | | 34.98 | | 35.00 | | 1.25 | |  |  |  |
| IL6 | 34.43 | | 31.36 | | 10.63 | |  | | TRAF3 | | 29.90 | | 29.94 | | 1.23 | |  |  |  |
| CXCL8 | 35.00 | | 31.83 | | 11.39 | |  | | TRAF6 | | 28.06 | | 28.64 | | -1.18 | |  |  |  |
| IRAK1 | 29.76 | | 30.84 | | -1.67 | |  | | TRIM25 | | 29.26 | | 28.46 | | 2.20 | |  |  |  |
| **En-Astrocytes** | | | | | | | | | | | | | | | | | | | |
| Gene symbol | | Ct | | | | Fold Regulation | |  | | Gene symbol | | Ct | | | | Fold Regulation | | |  |
|  |  | NI | | TBEV | |  |  |  | |  |  | NI | | TBEV | |  |  |  |  |
| AIM2 | | 32.78 | | 31.09 | | 4.00 | |  | | IRF3 | | 27.36 | | 27.38 | | 1.22 | | |  |
| APOBEC3G | | 35.00 | | 33.73 | | 2.99 | |  | | IRF5 | | 33.44 | | 35.00 | | -2.38 | | |  |
| ATG5 | | 26.55 | | 27.05 | | -1.14 | |  | | IRF7 | | 30.67 | | 28.58 | | 5.28 | | |  |
| AZI2 | | 26.23 | | 26.22 | | 1.25 | |  | | ISG15 | | 29.28 | | 22.87 | | 105.42 | | |  |
| CARD9 | | 35.00 | | 35.00 | | 1.24 | |  | | JUN | | 27.96 | | 27.51 | | 1.69 | | |  |
| CASP1 | | 30.97 | | 26.66 | | 24.59 | |  | | MAP2K1 | | 27.29 | | 27.87 | | -1.21 | | |  |
| CASP10 | | 33.70 | | 33.23 | | 1.72 | |  | | MAP2K3 | | 28.45 | | 28.39 | | 1.29 | | |  |
| CASP8 | | 31.26 | | 30.84 | | 1.66 | |  | | MAP3K1 | | 27.58 | | 28.16 | | -1.21 | | |  |
| CCL3 | | 35.00 | | 34.73 | | 1.49 | |  | | MAP3K7 | | 25.95 | | 26.48 | | -1.16 | | |  |
| CCL5 | | 35.00 | | 25.64 | | 814.63 | |  | | MAPK1 | | 24.56 | | 25.31 | | -1.36 | | |  |
| CD40 | | 35.00 | | 35.00 | | 1.24 | |  | | MAPK14 | | 27.32 | | 27.93 | | -1.23 | | |  |
| CD80 | | 35.00 | | 35.00 | | 1.24 | |  | | MAPK3 | | 27.69 | | 28.72 | | -1.65 | | |  |
| CD86 | | 35.00 | | 35.00 | | 1.24 | |  | | MAPK8 | | 26.28 | | 27.31 | | -1.65 | | |  |
| CHUK | | 28.14 | | 28.82 | | -1.29 | |  | | MAVS | | 27.88 | | 29.11 | | -1.89 | | |  |
| CTSB | | 26.03 | | 27.01 | | -1.59 | |  | | MEFV | | 35.00 | | 33.89 | | 2.68 | | |  |
| CTSL | | 26.01 | | 26.93 | | -1.53 | |  | | MX1 | | 32.82 | | 23.20 | | 975.50 | | |  |
| CTSS | | 34.59 | | 32.53 | | 5.17 | |  | | MYD88 | | 28.40 | | 26.81 | | 3.73 | | |  |
| CXCL10 | | 34.53 | | 24.60 | | 1209.34 | |  | | NFKB1 | | 30.02 | | 29.55 | | 1.72 | | |  |
| CXCL11 | | 35.00 | | 26.71 | | 388.02 | |  | | NFKBIA | | 28.45 | | 27.77 | | 1.99 | | |  |
| CXCL9 | | 30.31 | | 29.66 | | 1.95 | |  | | NLRP3 | | 35.00 | | 35.00 | | 1.24 | | |  |
| CYLD | | 28.55 | | 28.10 | | 1.69 | |  | | NOD2 | | 35.00 | | 35.00 | | 1.24 | | |  |
| TKFC | | 29.83 | | 31.33 | | -2.28 | |  | | OAS2 | | 35.00 | | 26.35 | | 498.00 | | |  |
| DDX3X | | 24.60 | | 25.46 | | -1.46 | |  | | PIN1 | | 27.35 | | 28.45 | | -1.73 | | |  |
| DDX58 | | 27.42 | | 23.73 | | 16.00 | |  | | PSTPIP1 | | 31.57 | | 33.28 | | -2.64 | | |  |
| DHX58 | | 35.00 | | 30.49 | | 28.25 | |  | | PYCARD | | 33.15 | | 33.43 | | 1.02 | | |  |
| FADD | | 28.60 | | 29.34 | | -1.35 | |  | | PYDC1 | | 35.00 | | 35.00 | | 1.24 | | |  |
| FOS | | 26.57 | | 27.07 | | -1.14 | |  | | RELA | | 27.43 | | 27.80 | | -1.04 | | |  |
| HSP90AA1 | | 22.74 | | 22.84 | | 1.16 | |  | | RIPK1 | | 29.06 | | 29.04 | | 1.26 | | |  |
| IFIH1 | | 28.84 | | 24.01 | | 35.26 | |  | | SPP1 | | 29.29 | | 28.68 | | 1.89 | | |  |
| IFNA1 | | 35.00 | | 35.00 | | 1.24 | |  | | STAT1 | | 26.05 | | 23.45 | | 7.52 | | |  |
| IFNA2 | | 35.00 | | 35.00 | | 1.24 | |  | | SUGT1 | | 25.27 | | 25.75 | | -1.13 | | |  |
| IFNAR1 | | 25.21 | | 26.35 | | -1.78 | |  | | TBK1 | | 27.69 | | 27.87 | | 1.09 | | |  |
| IFNB1 | | 35.00 | | 26.24 | | 537.45 | |  | | TICAM1 | | 30.38 | | 29.75 | | 1.92 | | |  |
| IKBKB | | 27.48 | | 28.17 | | -1.30 | |  | | TLR3 | | 29.49 | | 27.13 | | 6.36 | | |  |
| IL12A | | 29.11 | | 29.47 | | -1.04 | |  | | TLR7 | | 35.00 | | 35.00 | | 1.24 | | |  |
| IL12B | | 35.00 | | 35.00 | | 1.24 | |  | | TLR8 | | 35.00 | | 35.00 | | 1.24 | | |  |
| IL15 | | 34.26 | | 33.37 | | 2.30 | |  | | TLR9 | | 34.11 | | 35.00 | | -1.49 | | |  |
| IL18 | | 32.51 | | 33.22 | | -1.32 | |  | | TNF | | 35.00 | | 33.41 | | 3.73 | | |  |
| IL1B | | 34.50 | | 35.00 | | -1.14 | |  | | TRADD | | 32.70 | | 33.04 | | -1.02 | | |  |
| IL6 | | 34.78 | | 32.06 | | 8.17 | |  | | TRAF3 | | 28.27 | | 29.00 | | -1.34 | | |  |
| CXCL8 | | 35.00 | | 30.78 | | 23.10 | |  | | TRAF6 | | 27.44 | | 27.42 | | 1.26 | | |  |
| IRAK1 | | 29.35 | | 30.30 | | -1.56 | |  | | TRIM25 | | 26.87 | | 25.02 | | 4.47 | | |  |
